# DNA Damage: Quantum Mechanics/Molecular Mechanics Study on the Oxygen Binding and Substrate Hydroxylation Step in AlkB Repair Enzymes

**DOI:** 10.1002/chem.201303282

**Published:** 2013-12-11

**Authors:** Matthew G Quesne, Reza Latifi, Luis E Gonzalez-Ovalle, Devesh Kumar, Sam P de Visser

**Affiliations:** [a] Manchester Institute of Biotechnology and School of Chemical Engineering and Analytical Science, The University of Manchester131 Princess Street, Manchester M1 7DN (UK); [b] Department of Chemistry, Tufts UniversityMedford MA, 02155 (USA); [c] Department of Applied Physics, School of Physical Sciences, Babasaheb, Bhimrao Ambedkar UniversityVidya Vihar, Rae Bareilly Road, Lucknow 226-025 (India)

**Keywords:** density functional calculations, DNA base repair, DNA damage, hydroxylation, nonheme iron enzymes

## Abstract

AlkB repair enzymes are important nonheme iron enzymes that catalyse the demethylation of alkylated DNA bases in humans, which is a vital reaction in the body that heals externally damaged DNA bases. Its mechanism is currently controversial and in order to resolve the catalytic mechanism of these enzymes, a quantum mechanics/molecular mechanics (QM/MM) study was performed on the demethylation of the *N*^1^-methyladenine fragment by AlkB repair enzymes. Firstly, the initial modelling identified the oxygen binding site of the enzyme. Secondly, the oxygen activation mechanism was investigated and a novel pathway was found, whereby the catalytically active iron(IV)–oxo intermediate in the catalytic cycle undergoes an initial isomerisation assisted by an Arg residue in the substrate binding pocket, which then brings the oxo group in close contact with the methyl group of the alkylated DNA base. This enables a subsequent rate-determining hydrogen-atom abstraction on competitive σ-and π-pathways on a quintet spin-state surface. These findings give evidence of different locations of the oxygen and substrate binding channels in the enzyme and the origin of the separation of the oxygen-bound intermediates in the catalytic cycle from substrate. Our studies are compared with small model complexes and the effect of protein and environment on the kinetics and mechanism is explained.

## Introduction

Nonheme iron dioxygenases catalyse a range of important reactions in Nature including the biosynthesis of antibiotics in microbes and the metabolism of, for instance, cysteine in mammals.[1,2] In addition, nonheme iron dioxygenases have been linked to oxygen sensing and collagen cross-linking processes in the body, and as such they have vital functions for the biosystem,[3] but unfortunately there are many unanswered questions related to their activity and the catalytic transformation of substrates and detailed computational studies can shed light on this and predict a mechanism. The nonheme iron dioxygenases generally contain an iron active centre that is bound to the protein via a facial triad of amino acids that includes two histidine and one aspartate or glutamate residue in a 2-His/1-Asp feature.[4] They utilise a co-substrate (α-ketoglutarate, αKG) on an iron centre to convert molecular oxygen into a high-valent iron(IV)–oxo species, which has been proposed to be the active species of these enzymes.[5] For several nonheme iron dioxygenases, this active species has been characterised by spectroscopic methods, including resonance Raman and Mössbauer spectroscopies, and it was found that the iron(IV)–oxo intermediate reacts by hydrogen-atom abstraction from the substrate with a large kinetic isotope effect.[6] These experimental studies, however, mostly focused on the bacterial enzyme taurine/α-ketoglutarate dioxygenase.

A special class of enzymes within the nonheme iron dioxygenase family are the AlkB repair enzymes that repair methylated DNA (and RNA) bases that have been damaged by intra-or extracellular chemicals.[7] This has a dramatic effect on normal cellular function as, for instance, *N*^3^-methylation of an adenine residue of a DNA strain blocks DNA replication through the prevention of formation of Watson–Crick base-pairs.[8] In addition, chemicals such as methylmethane sulfonate and methyl halides have been shown to generate *N*^1^-methyladenine and *N*^3^-methylcytosine. AlkB repair enzymes are the body’s natural defence mechanism and react with damaged DNA and RNA chains by demethylation of these alkylated bases in a reaction that uses one molecule of αKG and molecular oxygen.[9] Biochemical studies were performed on the characterisation of the enzyme,[10] the protein structure,[11] and the substrate selectivity and binding of inhibitors.[12] Moreover, crystal structure coordinates were measured for the reactant as well as the substrate and hydroxylated product bound complexes.[13] These studies highlight a tight bonding pocket for substrate and a catalytic mechanism that leads to hydroxylation of the methylated DNA base.

**Figure 1:**
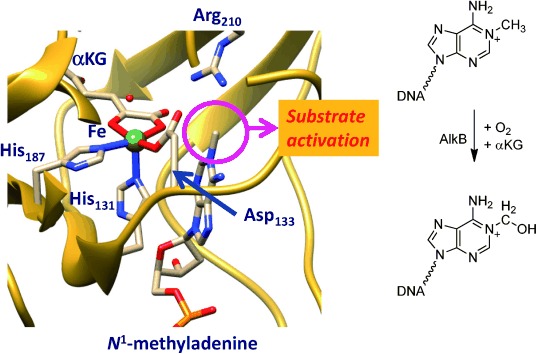
Extract of the active site of AlkB repair enzyme as taken from the 3I2O PDB file and the reaction catalysed by the enzyme. The proposed oxygen binding site *trans* to His_131_ is filled with a water molecule.

It has been hypothesised that the catalytic mechanism of AlkB repair enzymes is analogous to the nonheme iron dioxygenases and proceeds by aliphatic hydroxylation of the methyl group followed by release of formaldehyde.[7] Isotopic labelling and product distributions indeed confirmed that iron(II), αKG and molecular oxygen are needed in the process and that formaldehyde and CO_2_ are formed.[7a,b] Crystallographic data provided further evidence that AlkB repair enzymes belong to the αKG-dependent dioxygenases and highlighted a nonheme iron active site, where the metal is bound to a 2-His/1-Asp ligand system (Figure 1).[14] However, a close inspection of the crystal structure coordinates reveals that the sixth binding site of the metal, that is, the O_2_ binding site, is located perpendicular to the substrate orientation rather than in its vicinity. As such, an oxygen atom transfer within this conformation may be difficult and might proceed over a relatively large distance.

Furthermore, previous computational studies of the Gauld group on the catalytic cycle of the AlkB repair enzyme using active-site models found a rate-determining hydrogen-atom abstraction barrier of 20.9 kcal mol^−1^ for methylated adenine.[15] This is a relatively high barrier for a nonheme iron(IV)–oxo complex, since, for a series of hydrogen-atom abstraction reactions by analogous models much lower barriers were obtained for alternative substrates. As such, the value of 20.9 kcal mol^−1^ in the gas phase would compare to that found for a substrate like propane,[16] which as far as we know is not hydroxylated by nonheme iron enzymes. Consequently, a barrier with a magnitude over 20 kcal mol^−1^ may be a very slow process that is not efficient enough to happen in Nature. However, there may be effects of the protein and the local environment that were not taken into consideration in the models of Gauld et al. that have affected the barrier heights. The studies, therefore, warrant a further set of calculations and in particular one using quantum mechanics/molecular mechanics (QM/MM) that takes the full scale of the protein and solvent into effect.

**Scheme 1:**
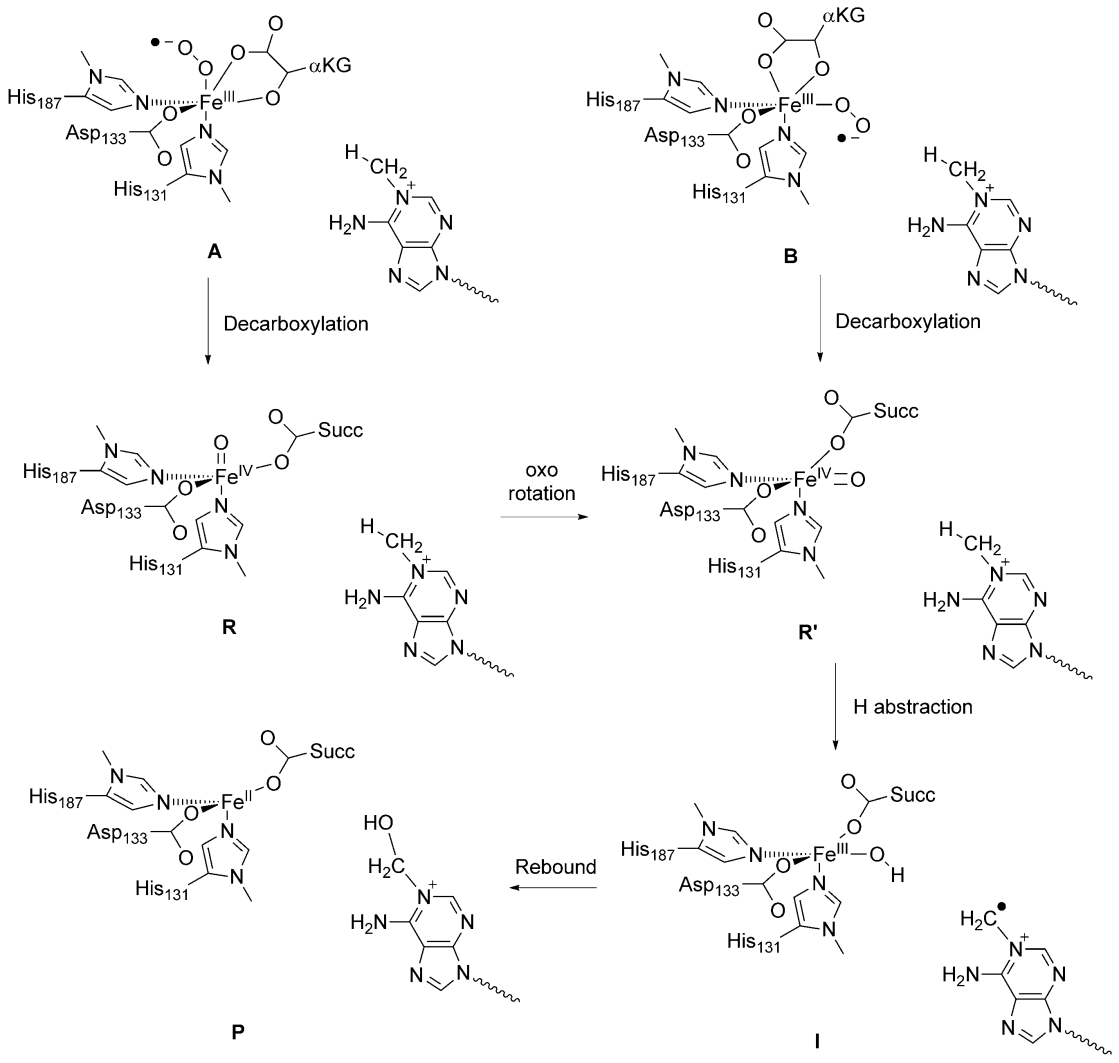
Mechanism of substrate hydroxylation by an iron(IV)–oxo in AlkB repair enzymes.

Here we report this QM/MM study and focus on the catalytic mechanism of oxygen activation by AlkB enzymes and the hydroxylation of methylated DNA bases (Scheme 1). We investigate two possible oxygen binding positions on the iron(II) reactant complex with the superoxo either *trans* to His_131_ (structure **A**) or *trans* to His_187_ (structure **B**). In the following step in the catalytic cycle the superoxo group is expected to attack the α-keto-position of αKG to give an iron(IV)–oxo species, CO_2_ and succinate.[17] Technically two isomeric iron(IV)–oxo structures are possible (**R** and **R′**), which may interconvert into each other. In this work we identify **A** as the oxygen-binding position and propose a novel mechanism whereby the iron(IV)–oxo undergoes an isomerisation from **R** to **R′** prior to hydrogen-atom abstraction. We also highlight the electronic changes during the reaction and the effect the isomerisation has on the electron-transfer pathways and the barrier heights for the reaction.

## Results and Discussion

### Oxygen binding site on the metal

A recent QM/MM study investigated the hydrogen-atom abstraction step of *N*^1^-methyladenine (CH_3_Ade) by AlkB repair enzymes.[18] They used a molecular geometry, whereby the oxo group was aligned with the substrate, that is, *trans* to His_187_, and an almost linear Fe-O-HCH_2_-Ade angle, analogous to structure **R′** in Scheme 1. However, the iron(IV)–oxo species is formed in a catalytic cycle from an iron(III)–superoxo complex in a reaction with αKG (Scheme 1), and in the crystal structure displayed in Figure 1 there seems to be a dioxygen binding site *trans* to His_131_. We decided, therefore, to first investigate the oxygen binding site of the enzyme, and thereby assign either **R** or **R′** as the reactant of the catalytic hydroxylation.

**Figure 2:**
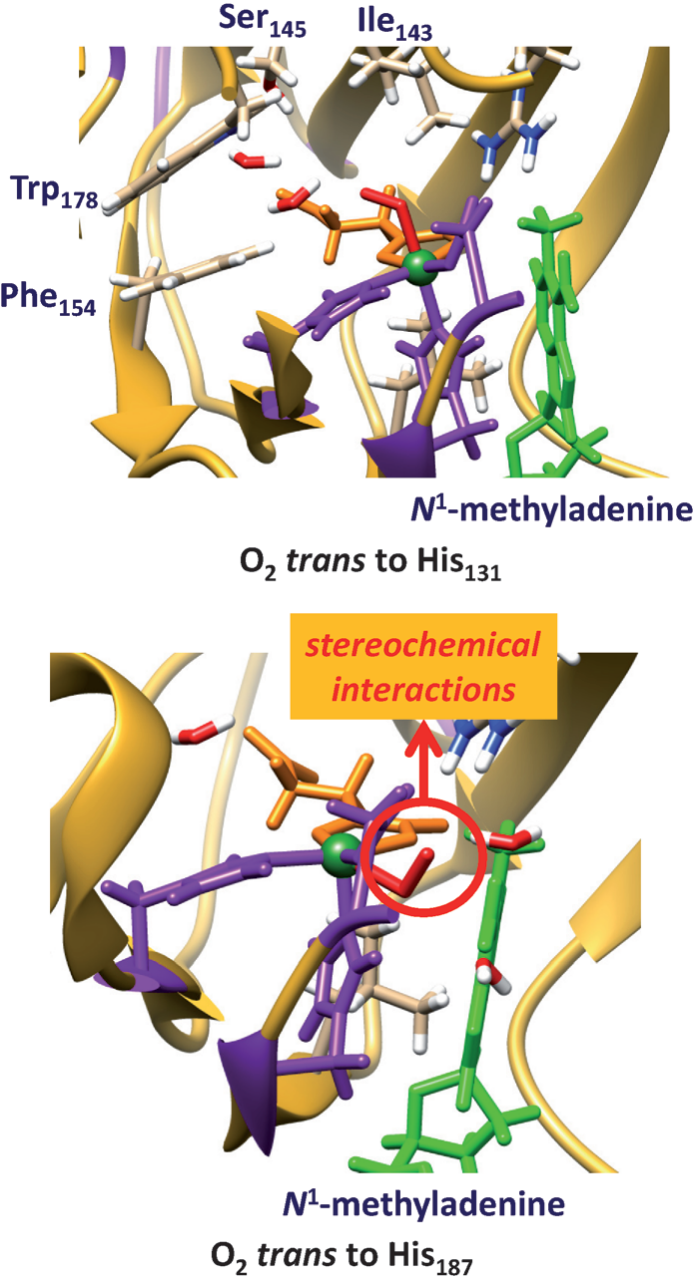
Isomeric structures for dioxygen binding to the iron(II) centre of AlkB enzymes with either the O_2_ *trans* to His_131_ (top) or *trans* to His_187_ (bottom). The former structure is MM minimised, whereas the latter failed to converge due to stereochemical clashes. The 2-His/1-Asp structure is in purple, substrate in green and αKG in orange.

We started the work with locating the oxygen binding site of AlkB, whereby we attempted to model dioxygen into the protein structure at various metal binding sites, that is, create structures **A** and **B** (Figure 2). Firstly, molecular oxygen was inserted into the sixth binding site *trans* to His_131_ by replacing the water ligand. The binding pocket has sufficient space to accommodate molecular oxygen and no stereochemical clashes are noted that would prevent it from binding in this position. The binding pocket is lined up with apolar and aromatic residues, such as the side chains of Ile_143_, Phe_154_ and Trp_178_. Solvation of the protein still finds sufficient space in the binding pocket *trans* to His_131_ for two water molecules; this binding pocket, therefore, is large enough to accommodate molecular oxygen. Figure 2 gives the equilibrated MM structure of **A**.

Thereafter, we inserted molecular oxygen into the metal position *trans* to His_187_ with the distal oxygen atom on the line through the iron and the hydrogen atom from *N*^1^-methyladenine that is abstracted by AlkB in order to generate structure **B**. However, this structure has considerable stereochemical constraints and we failed to optimise its geometry and converge it to a local minimum. In particular, the proximal oxygen atom of the iron(III)–superoxo with the oxygen *trans* to His_187_ gives close contacts (<1.7 Å) to the carboxylate group of Asp_133_ and the methyl group of *N*^1^-methyladenine. Furthermore, the distal oxygen atom of the iron(III)–superoxo has close contact with the carboxylate group of αKG (1.75 Å), therefore, stereochemically it is not a stable structure. Consequently, these MM models show that molecular oxygen does not fit into the binding pocket in a position *trans* to His_187_ as it is too tight with too many closely packed residues. As structure **B** is not a stable entity it cannot be a catalytic cycle intermediate and as a result molecular oxygen can only bind in the position *trans* to His_131_, that is, **A**. Our studies, therefore, have identified the molecular oxygen binding site as *trans* to His_131_ that in a reaction with αKG will give the iron(IV)–oxo species in the **R** conformation. This also implies that a subsequent isomerisation will be needed to form the rotated iron(IV)–oxo species (**R′**) with the oxo group *trans* to His_187_, which is expected to precede the hydrogen-atom abstraction reaction.

The reported QM/MM results in ref. [18] did not consider this isomerisation and as a result may not reflect the correct mechanism of the chemical reaction as they may have used a wrong starting structure of the chemical process. To gain further insight into the mechanism of substrate hydroxylation by DNA base repair enzymes, we decided to carry out a QM/MM study. We started the work from the iron(IV)–oxo species as displayed in Figure 1 and we followed the mechanism as described in Scheme 1 until hydroxylated products. The work started from the iron(IV)–oxo species with the oxo group *trans* to His_131_ (**R**), which is expected to isomerise to a position with the oxo group *trans* to His_187_ in **R′**. In the latter conformation the oxo group is in hydrogen bonding distance to the methyl group of *N*^1^-methyladenine and takes up a hydrogen atom to form a radical intermediate (**I**) before hydroxyl rebound to form alcohol product complexes (**P**).

### QM/MM set up and validation

The work described in here uses the quantum mechanics/molecular mechanics (QM/MM) procedure, whereby the inner core of the enzyme is described by density functional theory methods and the rest of the protein and solvent with a molecular mechanics force field as explained in detail in the Experimental Section. Generally, the methodology follows previous QM/MM calculations of our group that were carefully benchmarked and calibrated.[19] The calculations start from the 3I2O PDB file,[14] which is a substrate bound iron(III)–water complexed with αKG as described in Figure 1. The water ligand was replaced by an oxo group and αKG by succinate to obtain our reactant structure (**R′**): an iron(IV)–oxo group in a 2-His/1-Asp ligand environment and succinate bound as a bidentate ligand. Subsequently, hydrogen atoms were added and the protonation state of residues checked as described below. Thereafter, the structure was subjected to an iterative solvation procedure to give a chemical model with a total amount of 10 011 atoms.

**Figure 3:**
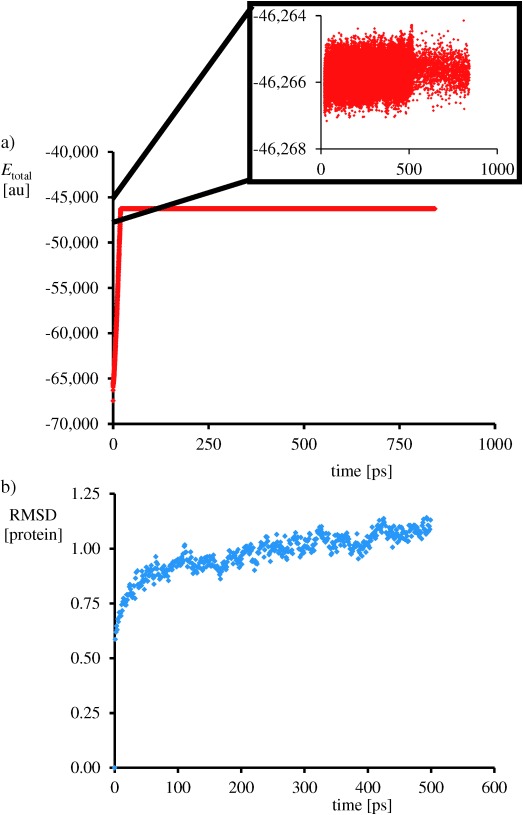
Molecular dynamics scan at 298 K of the protein. a) Plot of the total MM energy as a function of time. b) RMSD value of the protein structure as a function of time.

After the set-up was completed we ran a molecular dynamics (MD) simulation for 800 ps at 298 K and 1 bar using the Charmm force field,[20] whereby the protein and solvent were allowed to relax. Figure 3 displays the total energy and the root mean square deviation (RMSD) of the protein structure of the MD trajectory of our reactant structure. As follows from Figure 3 after 20–30 ps of simulation the total energy reaches a plateau and the energy stabilises. At the same time the fluctuations in the protein structure and geometry stabilise and the RMSD values converges to a value of close to 1. These MD simulations match those found for the analogous nonheme iron enzymes proline hydroxylase that revealed conformational stability and rigidity after substrate binding.[21]

**Figure 4:**
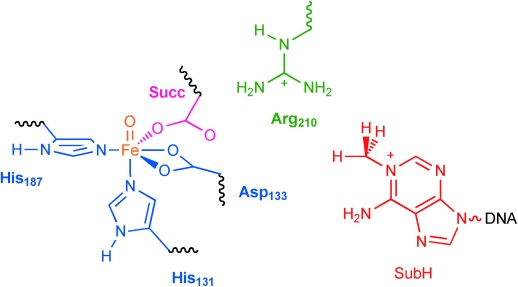
QM region selected for the QM/MM calculations. Waved lines represent QM/MM linkages that were described with the link atom method. Succ stands for succinate and SubH for substrate.

We selected three snapshots from the MD simulations as starting points for the QM/MM calculations after 300, 400 and 500 ps; designated Sn_300_, Sn_400_ and Sn_500_, respectively. An overlay of the three structures of these snapshots (Supporting Information, Figure S1) shows little differences in the protein orientation and the enzyme active site. The only differences obtained between these snapshots originate at distances far away from the reaction centre and generally are on the surface of the protein. We do not expect these motions and structural differences to be dramatic, but decided to carry out some test calculations for all three snapshots to determine the reproducibility and stability of the calculations. In all studies, we selected a large QM region containing the iron(IV)–oxo group, methylimidazole groups for His_131_ and His_187_, acetate for succinate and Asp_133_, methylguanidinium for Arg_210_, and the *N*^1^-methyladenine part of the substrate (Figure 4).

Subsequently, we optimised the geometry of the iron(IV)–oxo species using Sn_300_, Sn_400_ and Sn_500_ in the lowest lying singlet, triplet, quintet and septet spin states. In all cases, the geometry optimisation converged to a structure corresponding to **R′**, in which the oxo-group bridges between the methyl group of methylated adenine and the iron atom. The quintet spin state is the ground state in each snapshot and the spin state ordering follows quintet–triplet–septet and singlet throughout, which implies that the quintet spin state will be the reactive state. Previous studies of nonheme iron reactivities showed higher barriers on the triplet spin state than on the quintet spin state,[22] therefore, it is not expected that the triplet spin state will play a key role in this part of the catalytic cycle. Our calculations are in agreement with the experimental EPR characterisation of nonheme iron(IV)–oxo complexes in enzymes that were found to be in a high-spin state.[23] Previous calculations on the iron(IV)–oxo species of DNA base repair enzymes[15,18] and analogous nonheme iron dioxygenases[24] also identified it as a high-spin ground state. This contrasts biomimetic nonheme iron(IV)–oxo complexes that generally are described with a triplet spin ground state.[25] Recent computations, however, showed that pentacoordinated iron(IV)–oxo complexes stabilise the quintet spin state, whereas hexacoordinated iron(IV)–oxo usually has a triplet spin ground state.[26]

**Figure 5:**
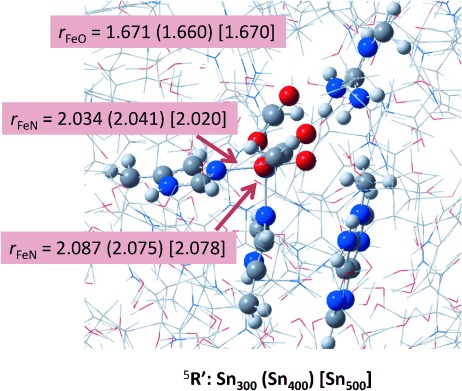
Optimised geometries of ^5^R′ as obtained for Sn_300_, Sn_400_ and Sn_500_ with bond lengths in Ångstroms.

All three snapshots give the same spin-state ordering and a well-separated quintet spin ground state from other states, therefore, we have focused in the following on the quintet spin mechanism of oxygen atom transfer only. Optimised geometries of ^5^**R′** as calculated in Sn_300_, Sn_400_ and Sn_500_ are given in Figure 5. The iron(IV)–oxo bond is short, that is, 1.660–1.671 Å, which is indicative of a double bond and in agreement with previous calculations on related complexes.[24,27] The structures show little deviations between the three snapshots, and consequently the QM region is highly rigid and constraint. Furthermore, the optimised geometries of the three snapshots implicates that the reproducibility of the results is high and that little movement in the central components of the protein and in particular the catalytic centre has occurred during the MD simulation. Further test calculations using different density functional methods were carried out, namely B3LYP, B3LYP*, BP86 and M06.[28]–[31] In all cases a spin state ordering quintet<triplet<singlet was found with the quintet spin state as the ground state (see the Supporting Information). As the DFT methodology does not appear to give dramatic differences in spin-state ordering and relative energies, we decided to continue with UB3LYP only.

### Iron(IV)–oxo group isomerisation

**Figure 6:**
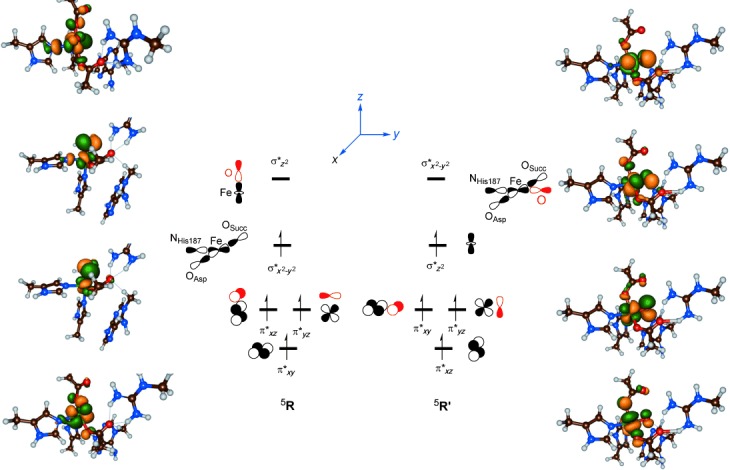
Orbital occupation of ^5^R and ^5^R′. Also given are the natural orbitals for ^5^R and ^5^R′.

In the next set of calculations, we investigated ^5^**R** as well as its barrier (^5^**TS**_I_) for the isomerisation into ^5^**R′**. A comparison of the structure and electronic properties of ^5^**R** and ^5^**R′** reveals some interesting features that may affect the reactivity of these complexes. Let us start with a description of the electronic changes upon rotation from ^5^**R** to ^5^**R′**. Thus, the rotation of the oxo group from a position *trans* to His_131_ in ^5^**R** to a position *trans* to His_187_ in ^5^**R′** changes the shapes of the molecular orbitals. Figure 6 displays the valence orbitals of ^5^**R** and ^5^**R′**, in which we used the nomenclature of Shaik, Solomon and co-workers for nonheme iron oxidants.[22] The labelling uses a molecular *z* axis as taken along the Fe—O bond in ^5^**R**. In the lowest quintet spin state of ^5^**R** the metal 3d block of orbitals interact with ligands and split into a set of three π* (π*_xz_, π*_yz_, π*_xy_) orbitals and a pair of two σ* (σ*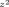, σ*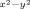) orbitals. The π* orbitals represent the antibonding combinations of the 3d atomic orbital on Fe with a 2p_*x*/*y*_ orbital on oxygen, whereas the σ*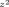 orbital reflects the antibonding combination of 3d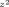 on Fe with 2p_*z*_ on O. Finally, the 3d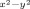 orbital on the metal forms antibonding combinations with ligands in the *xy* plane of symmetry. In pentacoordinated iron(IV)–oxo complexes the π*_xy_ and σ*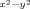 orbitals are close in energy and hence a high-spin situation is favourable.[32] Indeed, ^5^**R**, as found for analogous complexes,[15,18,19,24] has orbital occupation π*_xy_^1^ π*_xz_^1^ π*_yz_^1^ σ*
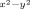
^1^ σ*
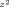
^0^. By contrast, the nearest triplet spin state is calculated to have π*_xy_^2^ π*_xz_^1^ π*_yz_^1^ configuration.

Upon rotation of the oxo group, whereby ^5^**R′** is formed the molecular orbital interactions have altered. Thus, the Fe—O bond is now located along the molecular *y* axis and hence the labels of some of the orbitals have changed. In particular, the π*_xy_ orbital in ^5^**R′** has a shape that matches the π*_xz_ orbital in ^5^**R** and the π*_xz_ orbital in ^5^**R′** looks like the π*_xy_ orbital in ^5^**R′**. However, since both are singly occupied in ^5^**R** and ^5^**R′**, that will not affect the relative energies of these isomers dramatically. The major differences between ^5^**R** and ^5^**R′** relate to the two σ* orbitals, that is, the HOMO and LUMO orbitals, which, therefore, also affect the reactivities. In ^5^**R** the σ*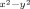 is singly occupied and the σ*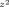 orbital is virtual, whereas the ordering is reversed for ^5^**R′** and the σ*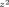 orbital is singly occupied instead. In this orientation the σ*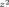 orbital in ^5^**R′** has lesser antibonding interactions than the HOMO, that is, σ*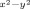 in ^5^**R** and consequently ^5^**R′** is more stable than ^5^**R**. We calculated an isomerisation energy difference of Δ*E*+ZPE=−6.0 kcal mol^−1^ for Sn_500_ at the UB3LYP level of theory. Changing the method to UB3LYP* gives negligible changes to the energy difference between ^5^**R** and ^5^**R′** and a value of −7.0 kcal mol^−1^ is obtained.

Despite the fact that the orbital interactions have changed between ^5^**R** and ^5^**R′**, there are actually very little changes in the group spin densities and charges of these complexes (see the Supporting Information). The spin density on the metal is slightly increased from 2.96 in ^5^**R** to 3.01 in ^5^**R′** and at the same time the oxygen atom loses spin density from 0.75 in ^5^**R** to 0.69 in ^5^**R′**. Generally, the more radical character obtained at the oxygen atom, the more reactive a metal–oxo group is, and it may be anticipated that this small change of spin polarisation from oxo to iron will make ^5^**R′** a slightly lesser oxidant than ^5^**R**. Our group spin densities and electronic state assignment matches those reported in previous DFT and QM/MM studies on analogous systems.[15–20]

In addition to differences in orbital interactions of the HOMO orbital in ^5^**R** versus ^5^**R′** there are similar changes noted for the LUMO orbital. In ^5^**R** the LUMO orbital is the antibonding interaction along the Fe—O bond, which shows little involvement of other ligands. By contrast, the σ*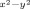 orbital displays interactions with four groups in the *xy* plane, namely the oxo, Asp_133_, His_187_ and succinate groups, and, therefore, it will be considerably higher in energy than the LUMO in ^5^**R**. Consequently, ^5^**R′** will have a much larger electron affinity than ^5^**R** and it will cost ^5^**R** more energy to abstract electrons from substrates. As hydrogen-atom abstraction is accompanied by a one-electron transfer from substrate to oxidant; this implies that ^5^**R′** will react with higher barriers than ^5^**R**. There must, therefore, be a fundamental reason for the enzyme to initiate the reaction with an isomerisation from ^5^**R** to ^5^**R′**. A possible reason is that the enzyme has to separate the dioxygen and substrate binding processes to avoid side reactions and by-products. Thus, after dioxygen binding to the iron centre an iron(III)–superoxo complex is formed that reacts with αKG to form an iron(IV)–oxo and succinate.[24] In several nonheme iron enzymes, however, the iron(III)–superoxo complex is known to abstract hydrogen atoms from substrates directly. For instance, in the enzyme isopenicillin N synthase, the tripeptide δ-(l-α-amino adipoyl)-l-cysteinyl-d-valine, by four sequential hydrogen-atom abstraction reactions, is converted into isopenicillin N through two ring-closure processes.[33] The first step of this reaction proceeds via an iron(III)–superoxo intermediate. It may very well be that in AlkB repair enzymes the iron(III)–superoxo has to be separated from substrate to prevent multiple hydroxylation reactions to occur on the methylated group, which would prevent the subsequent demethylation reaction into formaldehyde.

To test the relative reactivity of an iron(III)–superoxo versus an iron(IV)–oxo complex with *N*^1^-methyladenine, we set up a DFT model complex based on the QM region displayed in Figure 4 and calculated the hydrogen-atom abstraction of both complexes. We found a hydrogen-atom abstraction barrier of Δ*E*+ZPE=32.8 kcal mol^−1^ for the iron(III)–superoxo, whereas a value of Δ*E*+ZPE=23.4 kcal mol^−1^ for the iron(IV)–oxo was calculated. The latter matches the hydrogen-atom abstraction barrier reported by Gauld et al. well although slightly different methods were used.[15] Thus, the energy difference between the two hydrogen-atom abstraction barriers implicate faster reactivity of the iron(IV)–oxo than the iron(III)–superoxo complex. However, it is conceivable that the iron(III)–superoxo reacts with aliphatic groups by abstraction of hydrogen atoms although it probably will be slow.

Nevertheless, any reactivity of the iron(III)–superoxo species would lead to the disruption of the catalytic cycle and the failure to hydroxylate *N*^1^-methyladenine to form *N*^1^-hydroxymethyl adenine. It is, thus, very well possible that the enzyme has separated the oxygen binding and substrate binding positions in the active site of AlkB to prevent reactivity by the iron(III)–superoxo species. The substrate is shielded from the iron(III)–superoxo group by an arginine residue (Arg_210_) in the binding pocket. The superoxo group is too bulky to slide underneath Arg_210_ and attack the substrate, but once the iron(III)–superoxo has reacted with αKG and formed an iron(IV)–oxo species an oxidant is formed that can migrate to a position *trans* to His_187_ and attack the substrate. This way the enzyme retains the regioselectivity of substrate hydroxylation and prevents the occurrence of by-products. Most probably the enzyme pays a small thermodynamic price for this isomerisation process, which now happens with elevated hydrogen-atom abstraction barriers.

**Figure 7:**
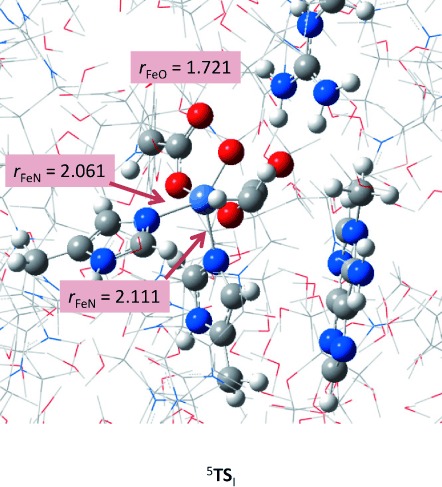
QM/MM optimised geometry of ^5^TS_I_ using snapshot Sn_500_ with bond lengths in Ångstroms.

Next we calculated the isomerisation barrier (^5^**TS**_I_) from ^5^**R** to ^5^**R′** in Sn_500_ and the optimised geometry is given in Figure 7. Energetically, ^5^**TS**_I_ is higher in energy than ^5^**R** by Δ*E*+ZPE=9.0 kcal mol^−1^. QM/MM studies of the Borowski group[34] on the nonheme iron halogenase SyrB2 were also predicted to start with an isomerisation, whereby the positions of the oxo and halide ligands to the metal centre were interchanged. They calculated an isomerisation barrier of 13.4 kcal mol^−1^ for that process, which is not dramatically different from the value we found here. Our isomerisation is slightly lower in energy because in AlkB only the oxo group migrates whereas in SyrB2 both the oxo and halide groups interchange, which will raise the barrier heights.

The optimised geometry of ^5^**TS**_I_ shows some differences with respect to ^5^**R** and ^5^**R′**. Obviously the NHis_131_-Fe-O angle changes along the rotation from 173.3° in ^5^**R** to 135.7° in ^5^**TS**_I_ and to 97.2° in ^5^**R′**. In addition in ^5^**TS**_I_ there is minor elongation of the two Fe—N distances with His_131_ and His_187_, but more dramatically is the lengthening of the Fe—O bond to 1.721 Å. This is because of a tight interaction of the oxo group with one of the protons of Arg_210_, which is shortened from 2.414 Å in ^5^**R** to 1.888 Å in ^5^**TS**_I_ and then elongates again toward ^5^**R′** to a value of 2.470 Å. The Arg_210_ residue, therefore, acts as a switch and assists with the rotation of the oxo group from *trans* to His_131_ to a position *trans* of His_187_.

### Substrate hydroxylation by the iron(IV)–oxo complex

We subsequently continued with calculations of the rest of the potential energy profile for the hydrogen-atom abstraction and followed by radical rebound to form alcohol product complexes (**P**). The hydrogen-atom abstraction passes a transition state (**TS**_H_) to form a radical intermediate (**I**) that is separated from products by a rebound transition state (**TS**_reb_). We calculated the hydrogen-atom abstraction with QM/MM using snapshot Sn_300_, Sn_400_ and Sn_500_, whereas the rebound was only investigated for Sn_500_. Despite the fact that we calculated the full potential energy profile of *N*^1^-methyladenine hydroxylation on the singlet, triplet, quintet and septet spin state surfaces with QM/MM, actually only the quintet spin state is accessible for this part of the catalytic cycle and the other spin states are much higher in energy (see the Supporting Information). This is in agreement with previous studies of nonheme iron(IV)–oxo complexes in which single-state reactivity on a dominant quintet spin state surface was found.[24a] Hence, we will focus on the quintet spin state results only and the other spin state structures and energetics are given in the Supporting Information for completeness.

**Figure 8:**
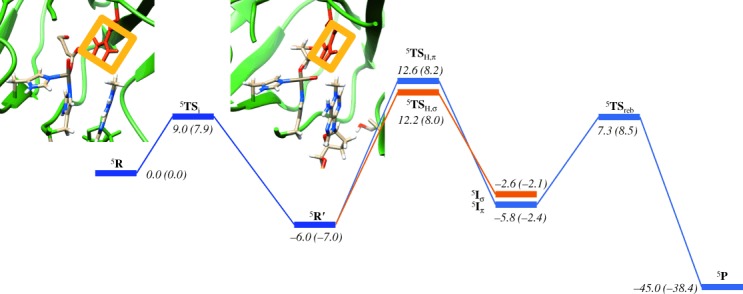
Potential energy landscape for the hydroxylation of *N*^1^-methyladenine as calculated with QM/MM. Energies are obtained at UB3LYP/B2//UB3LYP/B1-Amber, whereas in parenthesis are given UB3LYP*/B2//UB3LYP/B1-Amber results. All energies contain zero-point corrections and are given in kcal mol^−1^.

Figure 8 displays the complete potential energy profile from **R** to **P** as calculated with QM/MM in Sn_500_. As mentioned above, the isomerisation barrier is about 9.0 kcal mol^−1^ and leads to the energetically more stable structure **R′**. Geometrically, the isomerisation step is assisted by Arg_210_ that hydrogen bonds with the oxo and succinate groups. Arg_210_ keeps the oxo group in a specific orientation and guides its rotation toward the substrate. To highlight the motion of the Arg residue upon rotation from **R** to **R′** we have drawn a yellow box around its atoms. The isomerisation then brings the oxo group in close proximity to the substrate to enable a regioselective hydrogen-atom abstraction and prevent reactivity of its precursor the iron(III)–superoxo species. The tight substrate binding pocket and the Arg-assisted isomerisation mechanism enable regioselective hydroxylation of the methylated DNA base without activation of any of the other C—H bonds in the substrate.

We located two distinct pathways for hydrogen-atom abstraction by ^5^**R′**. Hydrogen-atom abstraction from *N*^1^-methyladenine by ^5^**R′** can either lead to electron transfer into the virtual σ*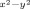 orbital, the so-called ^5^σ-pathway, or to double occupation of the π*_xz_ orbital through the so-called ^5^π-pathway.[35] Usually, in nonheme iron(IV)–oxo complexes the ^5^σ-pathway is considerably lower in energy than the ^5^π-pathway, and, hence is the dominating quintet spin electron transfer mechanism.[22,24,26] Generally, in the ^5^σ-pathway the substrate attacks from the top and incurs little stereochemical interactions with the metal ligands, whereas in the ^5^π-pathway the substrate approaches under an Fe-O-H angle of about 120° that is stereochemically disfavoured.[36] Due to the rotation of the iron(IV)–oxo group and a change of orbital shapes (Figure 6), the ^5^σ-pathway is not the dominating pathway anymore. We located transition states for both pathways, designated ^5^**TS**_H,σ_ and ^5^**TS**_H,π_, as well as the two isomeric radical intermediates, ^5^**I**_σ_ and ^5^**I**_π_.

Based on the orbital diagram for ^5^**R** and the unfavourable angle between the σ*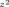 and the substrate location, one might have expected high barriers for ^5^**TS**_H,σ_ but actually due to the change of the ordering of σ*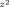 and σ*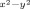 upon rotation of the iron(IV)–oxo group from ^5^**R** to ^5^**R′** the σ-pathway is still a viable reaction pathway. Moreover, the ^5^π-pathway is stabilised and becomes competitive with the ^5^σ-pathway. Nevertheless, the rate-determining step in the reaction mechanism in Figure 8 is the hydrogen-atom abstraction via barrier ^5^**TS**_H_, and both located transition states ^5^**TS**_H,σ_ and ^5^**TS**_H,π_ originate from the same reactant structure ^5^**R′**. Thus, during the hydrogen-atom abstraction an electron is transferred into the σ*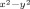 orbital in ^5^**TS**_H,σ_ to form an exchange coupled radical intermediate (^5^**I**_σ_) with π*_xz_^↑^ π*_yz_^↑^ π*_xy_^↑^ σ*
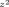
^↑^ σ*
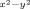
^↑^ ϕ_Sub_^↓^ configuration. By contrast in ^5^**TS**_H,π_ a low-lying π* orbital is doubly occupied to give a radical intermediate (^5^**I**_π_) with configuration π*_xz_^2^ π*_yz_^↑^ π*_xy_^↑^ σ*
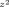
^↑^ σ*
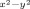
^0^ ϕ_Sub_^↑^. As the isomerisation has led to an inversion of the ordering of σ*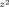 and σ*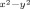 the σ-pathway now involves electron transfer into the σ*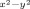 orbital and an alignment of the substrate along the molecular *y* axis. Indeed, an Fe-O-C angle of 141.4° is found for ^5^**TS**_H,σ_, whereas ^5^**TS**_H,π_ gives a much smaller angle of 131.4°.

Because of the fact that the angle in ^5^**TS**_H,σ_ deviates significantly from the ideal angle of a linear Fe-O-C conformation due to constraints on the enzyme substrate and co-factor binding pocket, the σ-pathway is destabilised. By contrast, the π-pathway requires angles of typically 120° and indeed here we find an Fe-O-C angle of 131.4°. Because of the unfavourable angle in the σ-pathway, it is destabilised in energy and the π-pathway becomes competitive. This is fundamentally different from hydrogen-atom abstraction along the molecular *z* axis as is normally the case for nonheme iron enzymes that appear to mostly react through the σ-pathway. Note as well that the LUMO orbital in ^5^**R**, that is, the σ*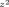 orbital is lower in energy than the LUMO orbital in ^5^**R′**, that is, the σ*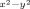 which has considerably more antibonding interactions between the metal and its ligands, and, therefore, it requires more energy to fill it.

Energetically in Sn_500_, ^5^**TS**_H,σ_ and ^5^**TS**_H,π_ are of comparable energies: 18.2 versus 18.6 kcal mol^−1^ at UB3LYP level of theory, and 15.0 versus 15.2 kcal mol^−1^ at UB3LYP* level of theory with respect to ^5^**R′**. We also calculated ^5^**R′** and ^5^**TS**_H,π_ in Sn_300_ and Sn_400_, but similar group spin densities and charges are found and a minor stabilisation of the barrier heights is observed: 13.0 kcal mol^−1^ for Sn_300_ and 15.1 kcal mol^−1^ for Sn_400_. As such there is a small effect of the protein on the barrier heights of the hydrogen-atom abstraction, although, geometrically they look very similar to the structure displayed in Figure 8 for Sn_500_ (see the Supporting Information). Our values are also close, but slightly lower in energy than those reported by Cisneros and co-workers.[18] Furthermore, DFT studies using an active-site complex found a rate-determining hydrogen-atom abstraction barrier of 20.9 kcal mol^−1^.[15] It appears, therefore, that the protein has a minor but stabilising effect on the transition state of the reaction. These high energetic barriers are caused through the isomerisation of the iron(IV)–oxo species, which raises the electron affinities and the corresponding hydrogen-atom abstraction barriers.

**Figure 9:**
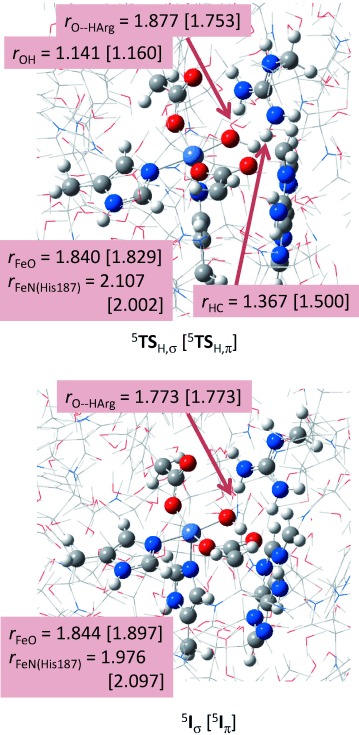
Optimised geometries of ^5^TS_H,σ_, ^5^TS_H,π_, ^5^I_σ_ and ^5^I_π_ as obtained from QM/MM calculations with bond lengths in Ångstroms.

Figure 9 gives QM/MM optimised geometries for hydrogen-atom abstraction transition states and intermediates on the competing σ-and π-pathways. In both cases the hydrogen-atom abstraction is late on the potential energy surface with short O—H distances of 1.141 and 1.160 Å for ^5^**TS**_H,σ_ and ^5^**TS**_H,π_, whereas their corresponding H—C distances are considerably longer: 1.367 and 1.500 Å, respectively. As previously shown,[37] late transition states generally correspond with high energetic barriers. Due to single occupation of the σ*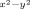 orbital in ^5^**TS**_H,σ_ the Fe—O and Fe—NHis_187_ distances are somewhat longer than those in ^5^**TS**_H,π_, in which this orbital is virtual. The Arg_210_ residue forms hydrogen bonding interactions with the carboxylate groups of succinate and Asp_133_ as well as with the oxo group at a distance 1.877 (1.753) Å for ^5^**TS**_H,σ_ (^5^**TS**_H,π_).

After the hydrogen-atom abstraction the system relaxes to a radical intermediate (^5^**I**_σ_ or ^5^**I**_π_), whereby the former has orbital occupation π*_xz_^↑^ π*_yz_^↑^ π*_xy_^↑^ σ*
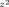
^↑^ σ*
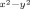
^↑^ ϕ_Sub_^↓^ whereas it is π*_xz_^2^ π*_yz_^↑^ π*_xy_^↑^ σ*
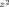
^↑^ σ*
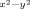
^0^ ϕ_Sub_^↑^ for the latter. The hydroxyl group in ^5^**I**_σ_ and ^5^**I**_π_ is locked in hydrogen-bonding interactions with the carboxylate group of Asp_133_ at a short distance of 1.701 (1.781) Å for ^5^**I**_σ_ (^5^**I**_π_). As such it is not surprising that hydroxyl rebound to the substrate radical gives a significant barrier: on the π-pathway we located a ^5^**TS**_reb_ barrier of 13.1 kcal mol^−1^ above ^5^**I**_π_, although this is lower in energy than the hydrogen-atom abstraction barrier of 18.6 kcal mol^−1^ for the π-pathway. This substantial rebound barrier was previously shown to lead to rearrangement patterns and the formation of by-products due to the relatively long lifetime of the radical intermediate.[38] Despite the large rebound barriers, therefore, the hydrogen-atom abstraction step is still the rate-determining step in the reaction mechanism. The large rebound barrier obtained in this work contrasts the result of DFT model calculations,[15] in which a rebound barrier of just 0.6 kcal mol^−1^ was found. Clearly, the strong hydrogen-bonding interactions of the OH group to the carboxylate groups of succinate and Asp_133_ and the methylguanidinium group of Arg_210_ stabilise the radical intermediates and raise the barrier for OH transfer to the radical. This highlights the importance of inclusion of part of the protein and particularly the hydrogen bonding network in the model.

**Figure 10:**
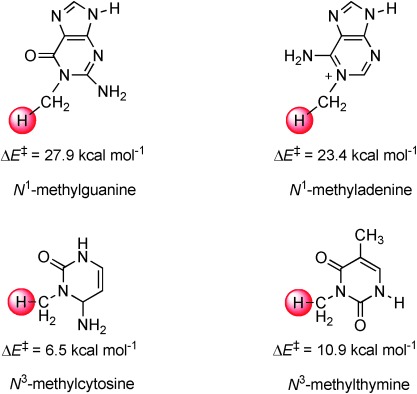
DFT calculated hydrogen-atom abstraction barriers from methylated DNA bases as calculated with DFT model complexes. All energies are in kcal mol^−1^ and contain ZPE and solvent corrections.

To find out whether the hydrogen abstraction barrier is dependent on the substrate, we also calculated it for *N*^1^-methylguanine, *N*^1^-methyladenine, *N*^3^-methylcytosine and *N*^3^-methylthymine using DFT model complexes and compared the results with the QM/MM studies described above (Figure 10). Our calculated hydrogen-abstraction barrier for *N*^1^-methyladenine of 23.4 kcal mol^−1^ is close in energy to the one reported by Gauld and co-workers.[15] We located hydrogen-atom abstraction barrier heights from the *N*-methyl positions of *N*^3^-methylcytosine, *N*^1^-methylguanine and *N*^3^-methylthymine of Δ*E*+ZPE=6.5, 27.9 and 10.9 kcal mol^−1^, respectively. To find out whether this trend correlates with the strength of the C—H bond that is broken for these substrates, we calculated the bond dissociation energy (BDE_CH_) for these bonds using procedures used before.[39] The BDE_CH_ value of substrates (SubH) was calculated from the energy difference of the isolated substrate with the sum of an isolated hydrogen atom and the substrate minus one hydrogen atom. As follows the BDE_CH_ values for these four substrates fall within a narrow window of about 5 kcal mol^−1^, and should have resulted in very similar barrier heights for all substrates. Inspection of the optimised geometries (see the Supporting Information) shows that in the DFT model complexes the substrates have reoriented in several cases and formed multiple hydrogen-bonding interactions with the carboxylic acid groups of succinate and Asp_133_ as well as the oxo-group. As shown before,[26] hydrogen-bonding interactions reduce catalytic efficiency and raise hydrogen-abstraction barriers and therefore these model complexes are not a good representative of the catalysis in the actual enzyme. Thermodynamically, the AlkB repair enzyme should, therefore, also be able to hydroxylate, and, consequently, repair *N*^3^-methylcytosine and *N*^3^-methylthymine.

## Conclusion

In this work we report a series of QM/MM and DFT studies on the catalytic mechanism of substrate activation by AlkB repair enzymes. We analysed PDB structures and attempted to insert a dioxygen molecule in several positions and found only one feasible binding position to iron *trans* to His_131_. The iron(III)–superoxo species is separated from the substrate by a considerable distance and its approach is blocked by an Arg residue. This Arg_210_ residue acts as a latch and only allows the isomerisation of iron(IV)–oxo and prevents the iron(III)–superoxo from reacting with substrate. It is proposed that the iron(III)–superoxo reacts with αKG to form iron(IV)–oxo, succinate and CO_2_. The iron(IV)–oxo initially has the oxo *trans* to His_131_ and isomerises to a position *trans* to His_187_. The isomeric iron(IV)–oxo species was found to react via hydrogen-atom abstraction on competing ^5^σ-and ^5^π-pathways to form a radical intermediate followed by rebound to give alcohol products. The hydrogen-atom abstraction is the rate-determining step in the reaction mechanism.

The studies presented in this work give important insight into the substrate and oxygen binding channels in the enzyme. We show that it is essential to separate the substrate and oxygen binding channels as otherwise the iron(III)–superoxo will react with substrate and prevent the repair reaction of the DNA. We also show that the isomerisation reorganises the high-lying occupied and low-lying virtual orbitals and, thereby, affects the electron transfer abilities of the oxidant. The isomeric iron(IV)–oxo reacts with substantially larger barriers as a result, which is a price the enzyme pays for separating the substrate and dioxygen binding channels.

## Experimental Section

For the set-up of the QM/MM system we used well-tested and benchmarked methods as reported before.[19] Starting from the 3I2O PDB file,[14] hydrogen atoms were added to the structure using the PDB2PQR program package[40] and the active site was manually modified from the iron(II)—water αKG complex into an iron(IV)–oxo succinate (Succ) active site. Apart from the two histidine groups that are bound to the metal, all other histidine side chains were doubly protonated. Furthermore, we made sure that all arginine and lysine side chains were protonated and all glutamic acid and aspartic acid side chains were deprotonated. This resulted in a structure with overall neutral charge. Solvent (with sphere of radius of 35 Å) was added to this structure, and equilibrated, followed by a molecular dynamics minimisation and heating procedure to 298 K of the full structure using the CHARMM force field.[20] The total model has 10 011 atoms and includes 2269 TIP3P water molecules. We selected several snapshots from this MD simulation as starting points for the QM/MM calculations at different time intervals. The iron(IV)–oxo reactant was geometry optimised in all low-lying and accessible spin states, that is, singlet, triplet, quintet and septet, and the spin state ordering and relative energies of these snapshots at 300, 400 and 500 ps (Sn_300_, Sn_400_, Sn_500_) gave consistent and reproducible results. Subsequently, we investigated the full potential energy profile with Sn_500_ as well as the hydrogen-atom abstraction step by Sn_300_ and Sn_400_. We chose to start the work from the iron(III)–superoxo complex and studied the mechanism until formation of alcohol product complexes as this procedure means the number of atoms in the model stays constant during the reaction.

QM/MM calculations employed the ONIOM program package as implemented in Gaussian 09.[41] The QM region was described by density functional theory and the unrestricted B3LYP functional,[28] while we used the Amber force field for the MM region.[42] The QM region contained the iron(IV)–oxo group, methylimidazole groups for His_131_ and His_187_, acetate for succinate and Asp_133_, methylguanidinium for Arg_210_, and the *N*^1^-methyladenine part of the substrate. All structures described here are the result of a full QM/MM geometry optimisation of all degrees of freedom. Stationary points were characterised by running an analytical frequency calculation on the QM region only at the same level of theory. Geometry optimisation and frequency were carried out with a double-ζ quality LACVP basis set on iron that contains a core potential and 6-31G on the rest of the atoms (basis set B1).[43] Single-point energy calculations with a Wachters all-electron basis set on iron and 6-31+G* on the rest of the atoms (basis set B2) was carried out on the optimised geometries at QM/MM to improve the energetics.[44] All energies reported in this work were obtained with basis set B2 and include ZPE corrections. Previous studies of our group on nonheme iron(IV)–oxo complexes showed these methods to be sufficiently accurate to match experimentally determined free energies of activation.[44] We used electronic embedding procedures whenever possible. To ascertain that the obtained results are reproducible we also calculated single-point energies using the UB3LYP* density functional in which the amount of HF exchange was reduced to 15 %,[29] as well as single points using either the BP86 or M06 methods.[30,31]

To gain further insight into the reaction kinetics and energetics we supplemented our studies with DFT model calculations in which we took a model containing iron(IV)–oxo with two imidazole and two acetate groups as the active species of the enzyme in analogy to previous model calculations.[16,24a] We then treated this oxidant with methylated DNA bases: *N*^1^-methyladenine, *N*^1^-methylguanine, *N*^3^-methylcytosine and *N*^3^-methylthymine. The geometries were optimised with UB3LYP/B1 and characterised with a frequency calculation. Single-point calculations were carried out with UB3LYP/B2 and solvent corrections were included.

We also calculated a DFT model complex based on the above-mentioned QM region for an iron(III)–superoxo complex and calculated the hydrogen-atom abstraction from *N*^1^-methyladenine at UB3LYP/B1. Geometries and frequencies were carried out in Gaussian using this level of theory and energies were corrected with single points at UB3LYP/B2 and solvent corrections.
