# Supplementary material for: DNA Damage: Quantum Mechanics/Molecular Mechanics Study on the Oxygen Binding and Substrate Hydroxylation Step in AlkB Repair Enzymes
Source: Chemistry. 2013 Dec 11;20(2):435–46. doi: 10.1002/chem.201303282 (PMC3994944; doi:10.1002/chem.201303282)
Supplement: Supplementary file 1 [file chem0020-0435-sd1.pdf]

CHEMISTRY
A EUROPEAN JOURNAL

Supporting Information

© Copyright Wiley-VCH Verlag GmbH & Co. KGaA, 69451 Weinheim, 2013

Quantum Mechanics/Molecular Mechanics Study on the Oxygen Binding and Substrate Hydroxylation Step in AlkB Repair Enzymes

Matthew G. Quesne,[a] Reza Latifi,[a, b] Luis E. Gonzalez-Ovalle,[a] Devesh Kumar,[c] and Sam P. de Visser*[a]

chem_201303282_sm_miscellaneous_information.pdf

Gaussian Reference

Gaussian 09, Revision **A.1**, Frisch, M. J.; Trucks, G. W.; Schlegel, H. B.; Scuseria, G. E.; Robb, M. A.; Cheeseman, J. R.; Scalmani, G.; Barone, V.; Mennucci, B.; Petersson, G. A.; Nakatsuji, H.; Caricato, M.; Li, X.; Hratchian, H. P.; Izmaylov, A. F.; Bloino, J.; Zheng, G.; Sonnenberg, J. L.; Hada, M.; Ehara, M.; Toyota, K.; Fukuda, R.; Hasegawa, J.; Ishida, M.; Nakajima, T.; Honda, Y.; Kitao, O.; Nakai, H.; Vreven, T.; Montgomery, Jr., J. A.; Peralta, J. E.; Ogliaro, F.; Bearpark, M.; Heyd, J. J.; Brothers, E.; Kudin, K. N.; Staroverov, V. N.; Kobayashi, R.; Normand, J.; Raghavachari, K.; Rendell, A.; Burant, J. C.; Iyengar, S. S.; Tomasi, J.; Cossi, M.; Rega, N.; Millam, J. M.; Klene, M.; Knox, J. E.; Cross, J. B.; Bakken, V.; Adamo, C.; Jaramillo, J.; Gomperts, R.; Stratmann, R. E.; Yazyev, O.; Austin, A. J.; Cammi, R.; Pomelli, C.; Ochterski, J. W.; Martin, R. L.; Morokuma, K.; Zakrzewski, V. G.; Voth, G. A.; Salvador, P.; Dannenberg, J. J.; Dapprich, S.; Daniels, A. D.; Farkas, Ö.; Foresman, J. B.; Ortiz, J. V.; Cioslowski, J.; Fox, D. J. Gaussian, Inc., Wallingford CT, 2009.

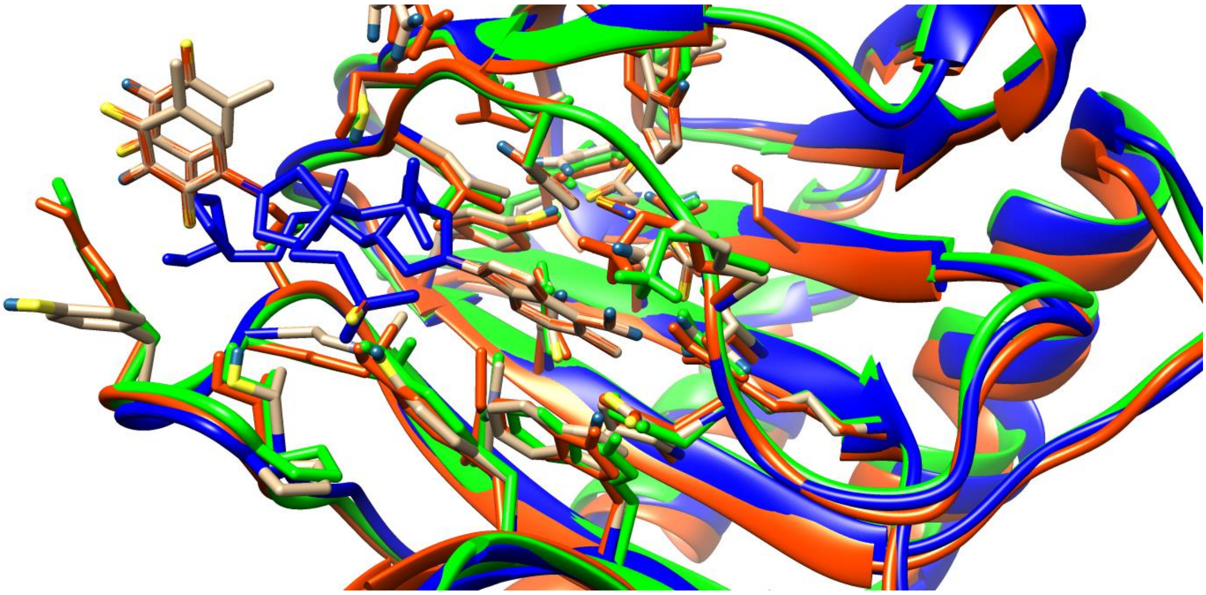

Figure S1. Overlay of the snapshots taken from the molecular dynamics simulation. Orange depicts the geometry at 300ps, green is that at 400ps whilst the blue colour represents the structure as seen after 500ps of MD simulation.

Table S1: A breakdown of the QM energies, in kcal mol^{-1}, reported on the quintet multiplicity of the 500ps snapshot. ^{5}**R** represents the original trans OXO geometry of the pdb structure, ^{5}**TS**$_{I}$ is the initial rotational barrier leading to the formation of the active cis OXO reactant ^{5}**R´**. ^{5}**TS**$_{H}$ indicates the limiting activation barrier for hydrogen abstraction from substrate leading to a radical intermediate **I**. The final rebound barrier ^{5}**TS**$_{reb}$ leads to the formation of the hydroxylated product and substrate demethylation.

	ΔE B3LYP/BS2	ΔE+ZPE B3LYP/BS2	ΔE B3LYP*	ΔE+ZPE B3LYP*	ΔE B3LYP/Amber	ΔE+ZPE B3LYP/Amber
^{5}**R**	0.00	0.00	0.00	0.00	0.00	0.00
^{5}**TS**$_{i}$	6.79	8.99	5.72	7.92	4.95	7.15
^{5}**R'**	-5.90	-5.98	-6.93	-7.01	-7.06	-7.14
^{5}**TS**$_{H,\pi}$	17.86	12.61	13.48	8.22	12.71	7.46
^{5}**I**$_{\pi}$	-4.73	-5.84	-1.34	-2.45	-6.04	-7.15
^{5}**TS**$_{reb}$	5.31	7.26	6.61	8.55	3.73	5.67
^{5}**P**	-49.27	-45.01	-42.65	-38.39	-50.51	-46.26
^{5}**TS**$_{H,\sigma}$	17.06	12.20	12.88	8.01	10.48	5.60
^{5}**I**$_{\sigma}$	-1.47	-2.58	-0.96	-2.07	-5.91	-7.02

Table S2: The spin state ordering of the QM/MM enzyme system, reported in kcal mol^{-1}. The values are relative to the quintet spin state of the 500ps snapshot. The spin state ordering is also related to ^{5}**R´** B3LYP/AMBER (BS2).

**Spin state ordering**	singlet	triplet	quintet	Septet
**R'**	39.14	17.54	0.00	17.62
**I_{π}**	64.47	12.44	1.17	4.58
**P**	-1.58	-18.18	-43.37	26.98

Table S3: The spin state ordering of the QM/MM enzyme system, reported in kcal mol^{-1}. The values are relative to the quintet spin states of each snapshot. The spin state ordering of the **R´** species are related to the quintet energies in B3LYP/AMBER (BS2).

**spin state ordering**	300 ps	400 ps	500 ps
singlet	32.58	34.66	39.14
triplet	7.66	9.90	17.54
quintet	0.00	0.00	0.00
septet	9.17	12.26	17.62

Table S4: Breakdown of the rate determining hydrogen abstraction barriers for the QM/MM AMBER:UB3LYP models taken at 300 and 400 picoseconds. All energies are given in kcal/mol^{-1} in relation to the ^{5}R' energies.

	B1 ΔE	B1 ΔE+ZPE	B2 ΔE	B2 ΔE+ZPE	B1 ΔG	B2 ΔG
**300 ps**						
^{5}R'	0.00	0.00	0.00	0.00	0.00	0.00
$^{5}TS_{H}$	18.98	11.33	20.66	13.01	11.12	12.80
**400 ps**						
^{5}R'	0.00	0.00	0.00	0.00	0.00	0.00
$^{5}TS_{H}$	23.14	15.31	22.92	15.09	13.38	13.16

Table S5: A breakdown of the spin density for the QM region of the 300, 400 & 500ps snapshots. Group spin densities were calculated for the iron centre (Fe), oxo group (OXO), succinate co-substrate (Succ) as well as the two histidine and one aspartate residues. Group spin densities in each case were assigned from the low spin singlet $^{1}R'$ through to the high spin septet $^{7}R'$.

Data for UB3LYP/BS2:

	Fe	OXO	Succ	SubH	HIS1	HIS2	ASP
**Snapshot 300**							
$^{1}R'$	0.00	0.00	0.00	0.00	0.00	0.00	0.00
$^{3}R'$	1.18	0.83	0.01	0.00	0.00	-0.04	0.01
$^{5}R'$	2.96	0.77	0.11	0.00	-0.01	0.10	0.07
$^{7}R'$	3.99	1.26	0.35	0.00	0.08	0.11	0.20
**Snapshot 400**							
$^{1}R'$	0.00	0.00	0.00	0.00	0.00	0.00	0.00
$^{3}R'$	1.70	0.35	-0.03	0.00	0.01	-0.05	0.02
$^{5}R'$	2.94	0.78	0.13	0.00	-0.01	0.08	0.08
$^{7}R'$	3.98	1.31	0.32	0.00	0.08	0.11	0.19
**Snapshot 500**							
$^{1}R'$	0.00	0.00	0.00	0.00	0.00	0.00	0.00
$^{3}R'$	1.68	0.37	-0.04	0.00	0.01	-0.04	0.02
$^{5}R'$	2.93	0.78	0.13	0.00	-0.01	0.08	0.08
$^{7}R'$	3.97	1.32	0.36	0.00	0.07	0.10	0.18

Table S6: Absolute energies (in au) for the QM/MM calculated structures. ZPE calculated on the QM region only.

	E(B3LYP), BS2 (au)	ZPE, BS1 (au)	E(B3LYP*), BS1 (au)	E(B3LYP), BS1 (au)
^{5}**R**	-1939.26792	0.40006	-1936.58802	-1938.77170
^{5}**TS**$_i$	-1939.25709	0.40356	-1936.57890	-1938.76381
^{5}**R'**	-1939.27732	0.39993	-1936.59906	-1938.78295
^{5}**TS**$_{H,\pi}$	-1939.23945	0.39169	-1936.56654	-1938.75144
^{5}**I**$_\pi$	-1939.27545	0.39829	-1936.59015	-1938.78133
^{5}**TS**$_{reb}$	-1939.25945	0.40316	-1936.57749	-1938.76576
^{5}**P**	-1939.34643	0.40684	-1936.65598	-1938.85220
^{5}**TS**$_{H,\sigma}$	-1939.24073	0.39229	-1936.56749	-1938.75500
^{5}**I**$_\sigma$	-1939.27026	0.39829	-1936.58955	-1938.78111

Table S7: The spin state ordering of the QM/MM enzyme system, reported in kcal mol^{-1}. The values are relative to the quintet spin state of the 500ps snapshot. The spin state ordering is also related to ^{5}**R´** and is recorder for AMBER combined to a variety of DFT methods, in BS2.

	singlet	triplet	quintet	septet
**B3LYP*/BS2**	31.11	10.81	0.00	17.46
**BP86/BS2**	22.65	5.54	0.00	33.71
**M06/BS2**	54.83	30.04	0.00	5.27

Table S8: A breakdown of the spin density for the QM region of the 500ps snapshot. $^{5}\mathbf{R}$ represents the original trans OXO geometry of the pdb structure, $^{5}\mathbf{TS}_{I}$ is the initial rotational barrier leading to the formation of the active cis OXO reactant $^{5}\mathbf{R'}$. $^{5}\mathbf{TS}_{H}$ indicates the limiting activation barrier for hydrogen abstraction from substrate leading to a radical intermediate, both the sigma and pi possibilities are shown **I**. The final rebound barrier $^{5}\mathbf{TS}_{reb}$ leads to the formation of the hydroxylated product and substrate demethylation.

Data for UB3LYP/BS1:

**BS1**	Fe	OXO	Succ	SubH	HIS1	HIS2	ASP
$^{5}\mathbf{R}$	2.84	0.86	0.14	0.00	0.10	0.00	0.06
$^{5}\mathbf{TS}_{I}$	2.82	0.88	0.12	0.00	0.08	0.07	0.03
$^{5}\mathbf{R'}$	2.93	0.78	0.13	0.00	-0.01	0.08	0.08
$^{5}\mathbf{TS}_{H,\pi}$	2.89	0.49	0.05	0.44	-0.02	0.09	0.06
$^{5}\mathbf{I}_{\pi}$	2.81	0.07	0.03	0.98	0.00	0.09	0.02
$^{5}\mathbf{TS}_{reb}$	3.11	-0.09	0.07	0.81	0.01	0.07	0.02
$^{5}\mathbf{P}$	3.74	0.00	0.13	0.00	0.05	0.05	0.04
$^{5}\mathbf{TS}_{H,\sigma}$	3.97	0.01	0.25	-0.53	0.09	0.10	0.10
$^{5}\mathbf{I}_{\sigma}$	4.07	0.32	0.31	-0.98	0.09	0.10	0.08

Table S9: A breakdown of the spin density for the QM region of the 500ps snapshot. ^{5}**R** represents the original trans OXO geometry of the pdb structure, ^{5}**TS**$_I$ is the initial rotational barrier leading to the formation of the active cis OXO reactant ^{5}**R´**. ^{5}**TS**$_H$ indicates the limiting activation barrier for hydrogen abstraction from substrate leading to a radical intermediate, both the sigma and pi possibilities are shown **I**. The final rebound barrier ^{5}**TS**$_{reb}$ leads to the formation of the hydroxylated product and substrate demethylation.
Data for UB3LYP/BS2:

**BS2**	Fe	OXO	Succ	SubH	HIS1	HIS2	ASP
^{5}**R**	2.96	0.75	0.13	0.00	0.09	0.00	0.07
^{5}**TS**$_I$	2.89	0.84	0.07	0.00	0.09	0.08	0.04
^{5}**R´**	3.01	0.69	0.14	0.00	-0.02	0.08	0.08
^{5}**TS**$_{H,\pi}$	3.52	0.14	0.06	0.07	0.03	0.11	0.05
^{5}**I**$_\pi$	2.82	0.07	0.03	0.99	-0.01	0.10	0.01
^{5}**TS**$_{reb}$	2.88	-0.01	0.05	0.96	0.00	0.09	0.03
^{5}**P**	3.77	0.00	0.10	0.00	0.05	0.06	0.03
^{5}**TS**$_{H,\sigma}$	4.08	-0.04	0.21	-0.51	0.08	0.09	0.10
^{5}**I**$_\sigma$	4.17	0.28	0.25	-0.98	0.10	0.10	0.08

Table S10: A breakdown of the spin density for the 500ps snapshot. ^{5}**R** represents the original trans OXO geometry of the pdb structure, ^{5}**TS**$_I$ is the initial rotational barrier leading to the formation of the active cis OXO reactant ^{5}**R´**. ^{5}**TS**$_H$ indicates the limiting activation barrier for hydrogen abstraction from substrate leading to a radical intermediate, both the sigma and pi possibilities are shown **I**. The final rebound barrier ^{5}**TS**$_{reb}$ leads to the formation of the hydroxylated product and substrate demethylation.
Data for UB3LYP*/BS1:

**B3LYP***	Fe	OXO	Succ	SubH	HIS1	HIS2	ASP
^{5}**R**	2.84	0.83	0.16	0.00	0.10	0.00	0.07
^{5}**TS**$_I$	2.85	0.85	0.10	0.00	0.08	0.07	0.04
^{5}**R´**	2.92	0.76	0.16	0.00	-0.01	0.08	0.09
^{5}**TS**$_{H,\pi}$	2.88	0.83	0.11	0.01	0.00	0.11	0.08
^{5}**I**$_\pi$	2.81	0.07	0.03	0.98	0.00	0.09	0.02
^{5}**TS**$_{reb}$	3.08	-0.07	0.12	0.77	0.01	0.06	0.02
^{5}**P**	3.71	0.00	0.14	0.00	0.05	0.05	0.05
^{5}**TS**$_{H,\sigma}$	3.88	0.06	0.25	-0.49	0.08	0.10	0.10
^{5}**I**$_\sigma$	4.04	0.33	0.32	-0.98	0.10	0.11	0.08

Data related to the DFT calculations of model complexes for hydroxylation of methylated cytosine (Cyt), thymine (Thy), guanine (Gua) and adenine (Ade). These structures were optimized without geometric constraints in Gaussian-09 and followed by a frequency calculation at UB3LYP/B1. A subsequent single point calculation used an LACV3P+ basis set on Fe and 6-311+G* on the rest of the atoms, basis set B3. Energies reported here, use the latter energy and include a correction for the zero-point energy as taken from the frequency calculations.

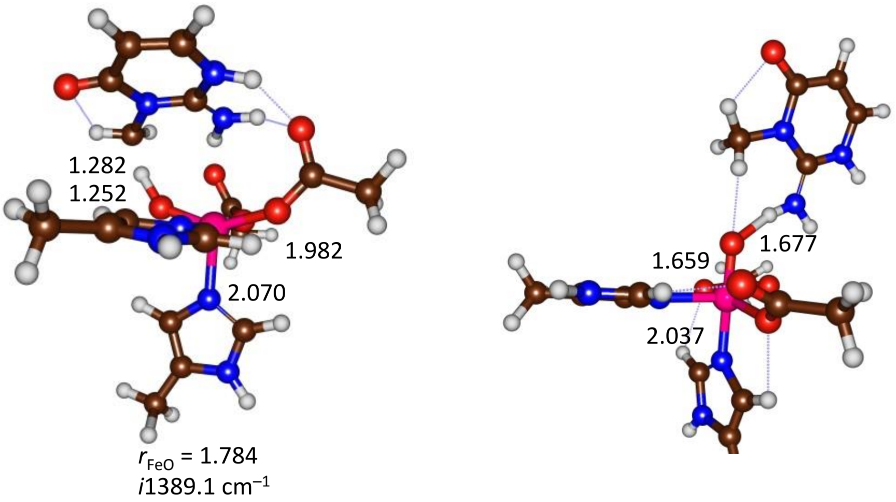

^{5}**TS**$_{H,Cyt}$ ^{5}**R**$_{Cyt}$

Figure S2. Optimized DFT geometries (UB3LYP/LACVP) of small active site model with distances in angstroms of the reactant complex and rate determining transition state of AlkB repair active site with **cytosine**. The imaginary frequency in the TS is given in wave numbers.

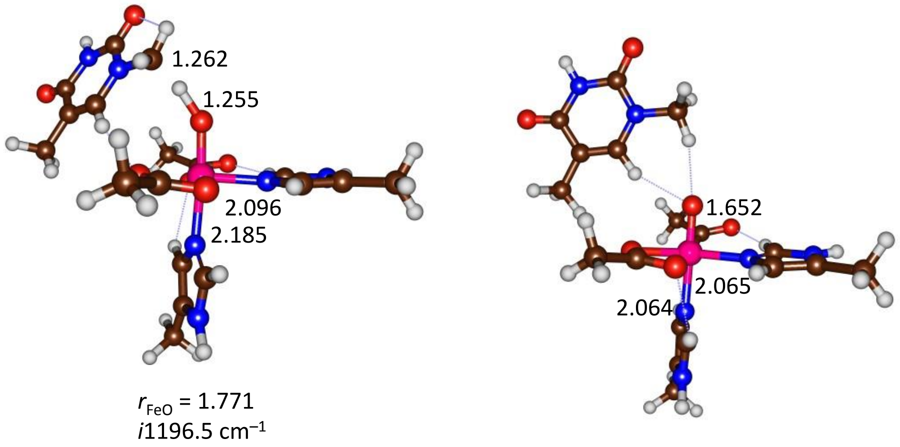

^{5}**TS**$_{H,Thy}$ ^{5}**R**$_{Thy}$

Figure S3. Optimized DFT geometries (UB3LYP/LACVP) of small active site model with distances in angstroms of the reactant complex and rate determining transition state of AlkB repair active site with **thymine**. The imaginary frequency in the TS is given in wave numbers.

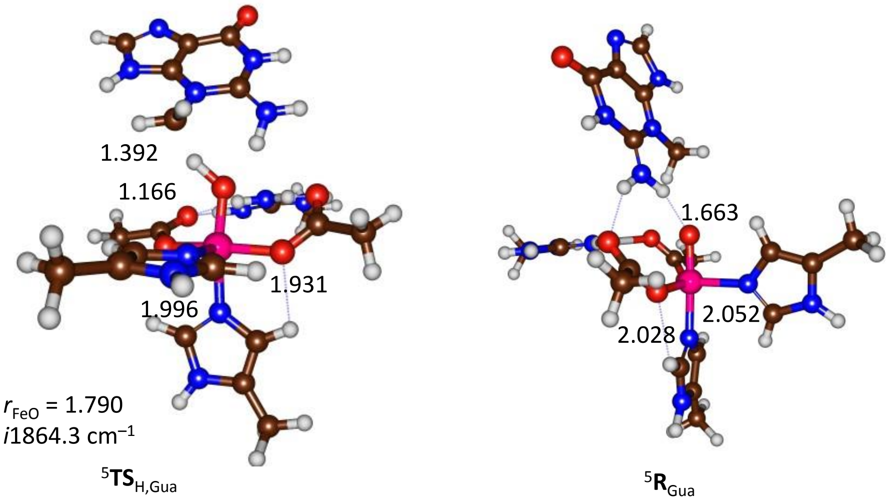

^{5}**TS**$_{H,Gua}$ ^{5}**R**$_{Gua}$

Figure S4. Optimized DFT geometries (UB3LYP/LACVP) of small active site model with distances in angstroms of the reactant complex and rate determining transition state of AlkB repair active site with **guanine**. The imaginary frequency in the TS is given in wave numbers.

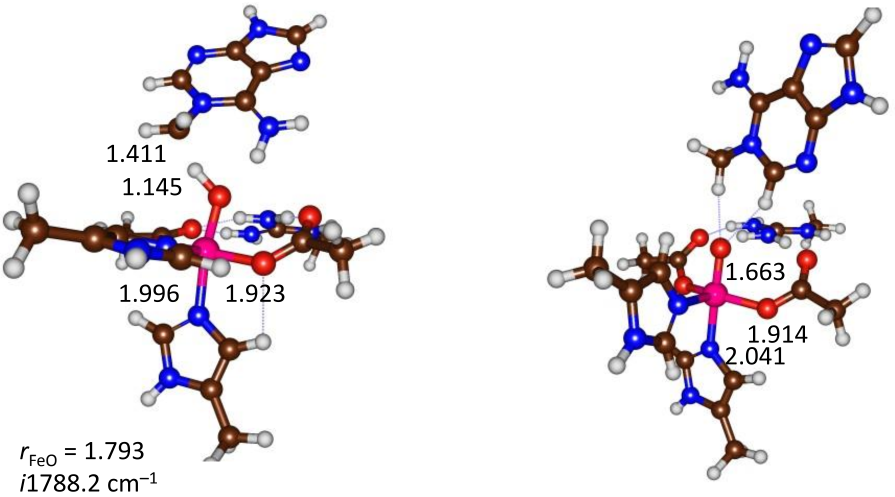

^{5}**TS**$_{H,Ade}$ ^{5}**R**$_{Ade}$

Figure S5. Optimized DFT geometries (UB3LYP/LACVP) of small active site model with distances in angstroms of the reactant complex and rate determining transition state of AlkB repair active site with **adenine**. The imaginary frequency in the TS is given in wave numbers.

Table S11: Group spin densities and charges of UB3LYP/B1 optimized geometries in Gaussian.

Spin	ρ_{Fe}	ρ_{O}	ρ_{His1}	ρ_{His2}	ρ_{Asp}	ρ_{Succ}	ρ_{SubH}
^{5}**R**$_{Cyt}$	3.07	0.60	0.08	-0.01	0.11	0.14	0.00
^{5}**TS**$_{H,Cyt}$	3.91	0.04	0.07	0.11	0.17	0.14	-0.45
^{5}**R**$_{Thy}$	3.00	0.69	0.05	-0.01	0.10	0.17	0.00
^{5}**TS**$_{H,Thy}$	3.90	0.09	0.08	0.05	0.14	0.18	-0.44
^{5}**R**$_{Gua}$	2.97	0.71	-0.01	0.10	0.11	0.13	-0.01
^{5}**TS**$_{H,Gua}$	2.86	0.42	-0.01	0.12	0.04	0.05	0.53
^{5}**R**$_{Ade}$	2.98	0.69	-0.01	0.10	0.11	0.13	0.00
^{5}**TS**$_{H,Ade}$	2.86	0.42	0.00	0.11	0.04	0.05	0.52

Charges	Q_{Fe}	Q_{O}	Q_{His1}	Q_{His2}	Q_{Asp}	Q_{Succ}	Q_{Succ}
^{5}**R**$_{Cyt}$	0.91	-0.45	0.21	0.28	-0.41	-0.44	0.89
^{5}**TS**$_{H,Cyt}$	1.14	-0.25	0.21	0.23	-0.45	-0.54	0.67
^{5}**R**$_{Thy}$	0.89	-0.36	0.18	0.23	-0.40	-0.50	-0.05
^{5}**TS**$_{H,Thy}$	1.04	-0.20	0.15	0.17	-0.48	-0.54	-0.14
^{5}**R**$_{Gua}$	1.04	-0.43	0.32	0.27	-0.48	0.41	0.87
^{5}**TS**$_{H,Gua}$	1.00	-0.24	0.97	0.23	-0.46	0.39	0.11
^{5}**R**$_{Ade}$	1.04	-0.42	0.30	0.25	-0.48	0.39	0.92
^{5}**TS**$_{H,Ade}$	0.99	-0.23	0.32	0.22	-0.46	0.41	0.75

Table S12: Group spin densities and charges of UB3LYP/B2//UB3LYP/B1 calculations in Gaussian.

Spin	ρ_{Fe}	ρ_{O}	ρ_{His1}	ρ_{His2}	ρ_{Asp}	ρ_{Succ}	ρ_{SubH}
^{5}**R**$_{Cyt}$	3.20	0.58	0.05	-0.08	0.11	0.14	-0.01
^{5}**TS**$_{H,Cyt}$	3.93	0.04	0.07	0.08	0.17	0.11	-0.40
^{5}**R**$_{Thy}$	3.12	0.68	0.02	-0.08	0.11	0.16	-0.01
^{5}**TS**$_{H,Thy}$	4.05	0.05	0.01	0.00	0.14	0.16	-0.41
^{5}**R**$_{Gua}$	3.10	0.69	-0.08	0.10	0.09	0.11	-0.01
^{5}**TS**$_{H,Gua}$	2.94	0.46	-0.06	0.09	0.03	0.04	0.50
^{5}**R**$_{Ade}$	3.15	0.67	-0.10	0.09	0.09	0.10	0.00
^{5}**TS**$_{H,Ade}$	2.95	0.47	-0.07	0.09	0.03	0.03	0.50

Charge	Q_{Fe}	Q_{O}	Q_{His1}	Q_{His2}	Q_{Asp}	Q_{Succ}	Q_{Succ}
^{5}**R**$_{Cyt}$	-2.60	0.76	1.06	0.68	0.04	0.20	0.86
^{5}**TS**$_{H,Cyt}$	-1.79	0.44	0.86	0.85	0.25	0.26	0.14
^{5}**R**$_{Thy}$	-2.80	0.89	1.03	0.50	0.06	0.35	-0.03
^{5}**TS**$_{H,Thy}$	-2.64	0.71	1.18	1.00	-0.29	0.37	-0.31
^{5}**R**$_{Gua}$	-2.43	0.29	1.21	2.29	-0.42	2.32	-1.24
^{5}**TS**$_{H,Gua}$	-3.50	0.79	1.00	1.40	0.14	1.41	0.76
^{5}**R**$_{Ade}$	-2.74	0.54	0.82	1.07	0.16	1.19	0.95
^{5}**TS**$_{H,Ade}$	-3.83	0.74	1.16	1.52	0.22	1.42	0.78

Table S13: Absolute energies (in au) for UB3LYP/B1 optimized geometries in Gaussian.

	E, au<br>LACVP	ZPE, au<br>LACVP	G, au<br>LACVP	E, au<br>LACV3P+*	Esolv, au<br>LACV3P+*
$^{5}\mathbf{R}_{\mathrm{Cyt}}$	-1621.395603	0.450904	-1621.018262	-1621.856243	-1621.467341
$^{5}\mathbf{TS}_{\mathrm{H,Cyt}}$	-1621.386894	0.444186	-1621.017510	-1621.839243	-1621.450297
$^{5}\mathbf{R}_{\mathrm{Thy}}$	-1680.183535	0.452950	-1679.809764	-1680.674030	-1680.228574
$^{5}\mathbf{TS}_{\mathrm{H,Thy}}$	-1680.160597	0.445429	-1679.791831	-1680.649063	-1680.203073
$^{5}\mathbf{R}_{\mathrm{Gua}}$	-2014.055439	0.589636	-2013.552856	-2014.630435	-2014.241848
$^{5}\mathbf{TS}_{\mathrm{H,Gua}}$	-2014.006829	0.582484	-2013.511709	-2014.578737	-2014.202472
$^{5}\mathbf{R}_{\mathrm{Ade}}$	-1938.831416	0.585601	-1938.335761	-1939.385474	-1939.003789
$^{5}\mathbf{TS}_{\mathrm{H,Ade}}$	-1938.792231	0.578550	-1938.299071	-1939.341204	-1938.965768

Table S14: Relative energies and free energies of UB3LYP/B1 optimized geometries in Gaussian. Values in kcal/mol.

	ΔE<br>B1	ΔE+ZPE<br>B1	ΔG<br>B1	ΔE<br>B2	ΔE+ZPE<br>B2	ΔG<br>B2	ΔE+ZPE+Es<br>B2	ΔG+Es<br>B2
$^{5}\mathbf{R}_{\mathrm{Cyt}}$<br>$^{5}\mathbf{TS}_{\mathrm{H,Cyt}}$	5.47	1.25	0.47	10.67	6.45	5.67	6.48	5.70
$^{5}\mathbf{R}_{\mathrm{Thy}}$<br>$^{5}\mathbf{TS}_{\mathrm{H,Thy}}$	14.39	9.67	11.25	15.67	10.95	12.53	11.28	12.86
$^{5}\mathbf{R}_{\mathrm{Gua}}$<br>$^{5}\mathbf{TS}_{\mathrm{H,Gua}}$	30.50	26.02	25.82	32.44	27.95	27.76	20.22	20.03
$^{5}\mathbf{R}_{\mathrm{Ade}}$<br>$^{5}\mathbf{TS}_{\mathrm{H,Ade}}$	24.59	20.16	23.02	27.78	23.36	26.21	19.43	22.29

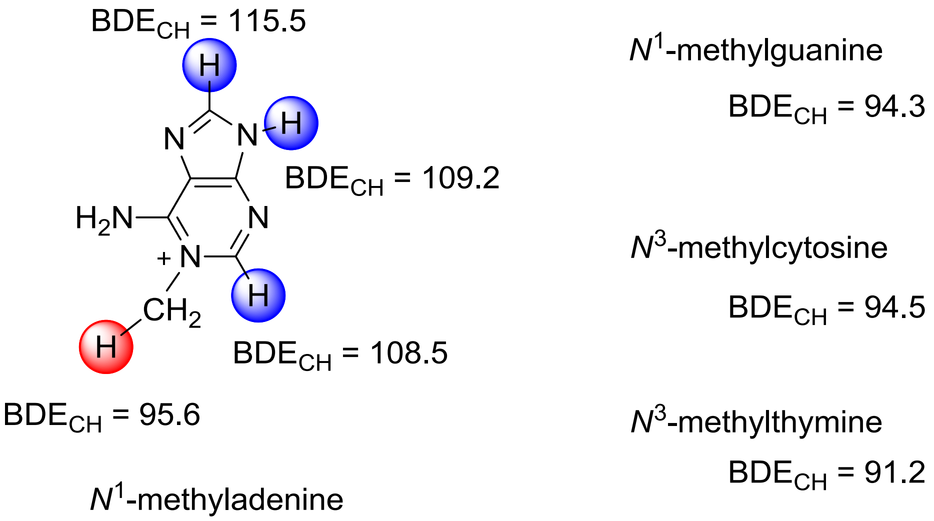

Figure S6. DFT calculated bond strengths (BDE$_{C–H}$) at the UB3LYP/B2+ZPE//UB3LYP/B1 level of theory of methylated DNA bases. All values are in kcal mol^{-1}.

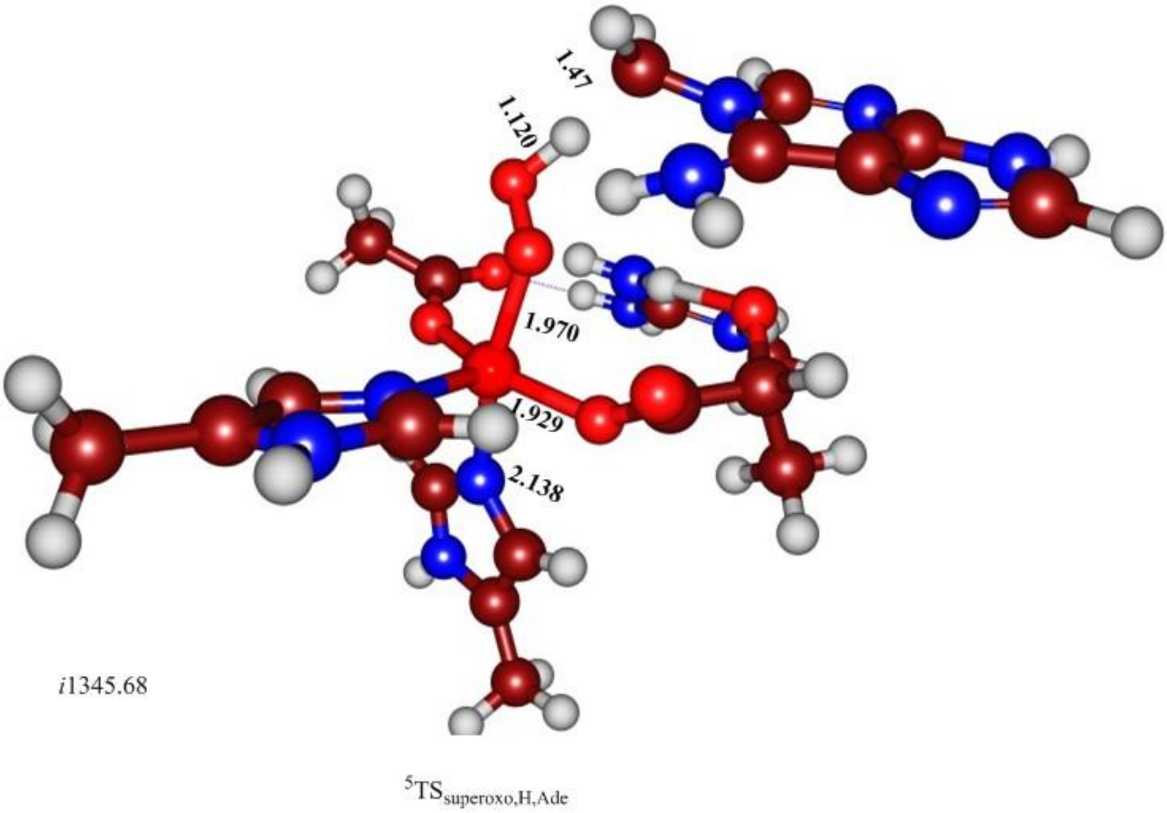

Figure S7. Optimized DFT superoxo transition state geometry (UB3LYP/LACVP) of small active site model species with distances in angstroms. The substrate for the AlkB repair active site is **adenine** and the imaginary frequency is given in wave numbers.

Table S15: Breakdown of the relative energies of the model superoxo system taken from the coordinates of the QM/MM ^{5}R species. The barrier for superoxo hydrogen abstraction is calculated using UB3LYP/LACVP and all energies are related to ^{5}Re. Values are given in Kcal Mol-1 and the solvent added using the *Gaussian 09* PCM method with a ε=5.697.

**DNA superoxo**	B1 ΔE	B1 ΔE+ZPE	B2 ΔE	B2 ΔE+ZPE	B1 ΔG	B2 ΔG	B2 +Esolv ΔE+ZPE	B2 +Esolv ΔG
**^{5}R’**	0.00	0.00	0.00	0.00	0.00	0.00	0.00	0.00
**$^{5}TS_{H}$**	33.87	30.27	36.39	32.78	33.78	36.30	43.45	46.96
**^{5}I**	23.96	22.96	27.33	26.33	25.86	29.23	22.56	25.46

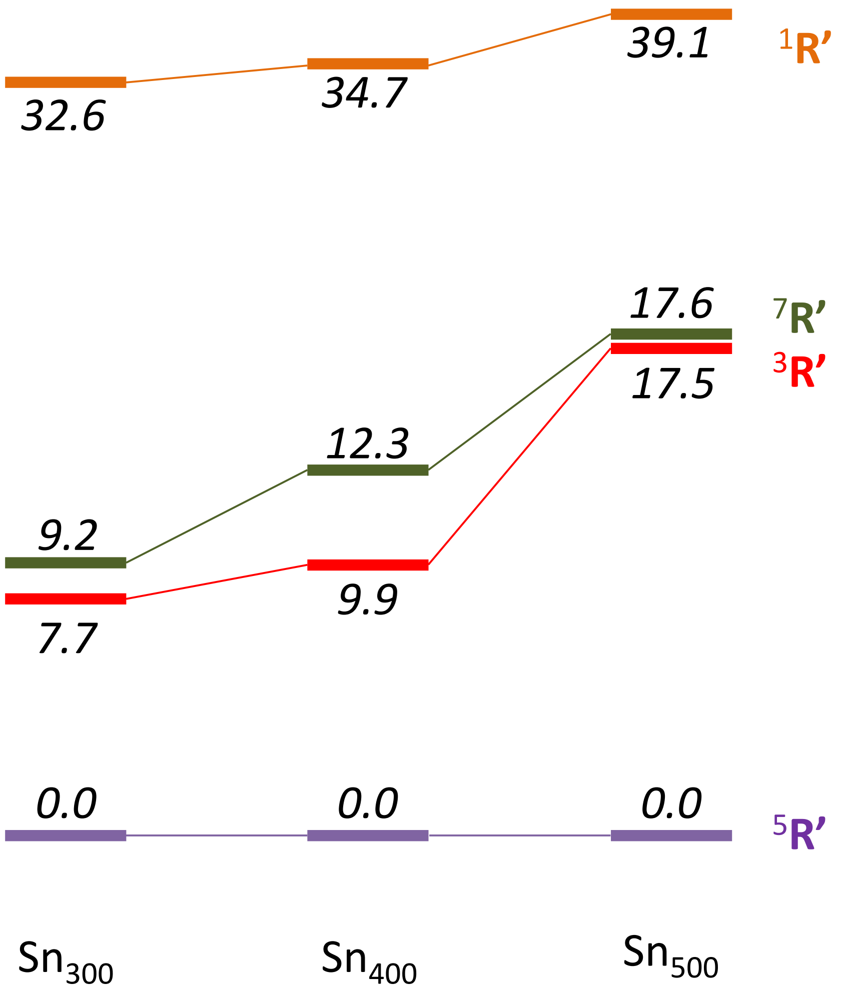

Figure S8: Spin state ordering and relative energies (in kcal/mol) of structure **R‘** as obtained in three different snapshots.

Cartesian coordinates of all structures described in this work:
QM region of QM/MM calculations:

^{5}R

6	-0.113468000	1.852096000	-5.349602000
1	-0.406510000	1.270596000	-6.226445000
1	-0.860281000	2.631340000	-5.215005000
7	0.669356000	-0.198394000	-4.043411000
1	1.122783000	-0.645479000	-4.820115000
6	-0.039733000	1.008014000	-4.108510000
6	0.662246000	-0.654173000	-2.766275000
1	1.112162000	-1.557677000	-2.401602000
7	-0.037462000	0.183843000	-2.000632000
6	-0.483225000	1.220593000	-2.829051000
1	-1.066166000	2.022850000	-2.424215000
6	-1.901994000	3.738488000	1.300069000
1	-2.901240000	4.041544000	1.610391000
1	-1.222484000	3.885929000	2.143944000
6	-1.876000000	2.272179000	0.919507000
8	-1.052682000	1.852305000	0.000827000
8	-2.644220000	1.463303000	1.544663000
6	5.487343000	1.482312000	-0.864019000
1	6.081764000	2.126986000	-0.210162000
1	5.838416000	0.461248000	-0.721180000
7	3.275332000	2.731134000	-0.622167000
1	3.651377000	3.638052000	-0.837537000
6	4.021095000	1.555411000	-0.558409000
6	1.968813000	2.452270000	-0.367116000
1	1.160393000	3.156601000	-0.364195000
7	1.844061000	1.149312000	-0.112711000
6	3.110978000	0.581280000	-0.230290000
1	3.254242000	-0.470940000	-0.075488000
6	-4.837887000	0.484141000	6.455228000
1	-5.870685000	0.391683000	6.112410000
1	-4.759097000	0.000679000	7.424641000
7	-3.891970000	-0.180652000	5.534814000
1	-3.392415000	-0.978246000	5.888923000
6	-3.585291000	0.233342000	4.286010000
7	-4.269467000	1.252050000	3.723051000
1	-5.060774000	1.677825000	4.165236000
1	-3.881473000	1.609555000	2.836126000
7	-2.593637000	-0.331090000	3.589310000
1	-1.980738000	-1.042706000	3.955608000
1	-2.277110000	0.059631000	2.684772000
26	0.013998000	0.176563000	0.031634000
8	-0.076820000	0.145286000	1.695769000
6	1.542419000	-3.666648000	0.765298000
1	1.238878000	-4.200680000	-0.136813000
1	2.552333000	-3.285935000	0.596086000
6	0.582994000	-2.524999000	1.030774000
8	-0.077554000	-2.429325000	2.075697000
8	0.476389000	-1.666541000	-0.020864000
7	-3.364354000	-0.083706000	-5.350275000
6	-5.064310000	0.634632000	-0.187606000
1	-5.479459000	-0.293025000	0.203180000
1	-5.825583000	1.411443000	-0.160036000
1	-4.212664000	0.926617000	0.421788000
6	-4.046550000	1.201894000	-3.717933000
7	-3.801035000	2.052492000	-4.782644000
6	-3.372987000	1.251561000	-5.751243000
1	-3.105856000	1.551945000	-6.746712000
7	-4.676452000	0.417046000	-1.599632000
6	-4.396635000	-0.878096000	-2.030530000
1	-4.538389000	-1.653676000	-1.294833000
7	-3.971340000	-1.198698000	-3.228936000
6	-3.784528000	-0.127507000	-4.049610000
6	-4.516916000	1.501079000	-2.435439000
7	-4.809304000	2.749147000	-2.059562000
1	-5.235917000	3.339876000	-2.758389000
1	-4.166202000	3.269868000	-1.467822000
1	5.706243000	1.765698000	-1.897876000
1	0.847046000	2.328834000	-5.569398000
1	-3.097954000	-0.880643000	-5.901017000
1	1.554045000	-4.354825000	1.608540000
1	-4.588168000	1.539292000	6.577692000
1	-1.563346000	4.365623000	0.477169000

$^{5}TS_{I}$

7	2.492426000	-1.000200000	2.914348000
1	2.534242000	-1.481232000	3.794985000
6	3.426325000	-0.077972000	2.460123000
6	1.514345000	-1.126485000	1.996710000
1	0.641321000	-1.745569000	2.084600000
7	1.771869000	-0.364887000	0.948071000
6	2.960132000	0.296875000	1.229930000
1	3.379050000	0.987317000	0.525229000
6	1.731648000	1.953401000	-2.354183000
8	1.469103000	1.634704000	-1.132453000
8	1.561256000	1.191692000	-3.321731000
7	-0.292033000	3.376931000	2.262107000
1	0.056534000	4.215191000	2.692438000
6	-1.351288000	2.620950000	2.732359000
6	0.212445000	2.797559000	1.159380000
1	1.050396000	3.149128000	0.590791000
7	-0.484351000	1.712306000	0.857687000
6	-1.453200000	1.591442000	1.839950000
1	-2.124873000	0.756228000	1.835982000
7	-0.630212000	0.008346000	-7.042681000
1	-1.480308000	-0.519764000	-6.946882000
6	0.126729000	0.159241000	-5.944650000
7	1.316991000	0.777456000	-6.027347000
1	1.715500000	1.065761000	-6.899198000
1	1.722043000	1.079672000	-5.130941000
7	-0.297494000	-0.267510000	-4.760253000
1	-1.232044000	-0.625331000	-4.635485000
1	0.162205000	-0.089997000	-3.832116000
26	0.263948000	0.235597000	-0.449429000
8	-0.111801000	-0.269580000	-1.972633000
6	-2.164250000	-1.223580000	-0.303769000
8	-2.584278000	-0.625990000	-1.272385000
8	-0.994317000	-0.978076000	0.290574000
7	5.706038000	-2.984372000	1.162877000
6	3.669918000	-1.047277000	-3.499730000
1	3.251978000	-1.856301000	-4.095451000
1	4.479759000	-0.567221000	-4.043021000
1	2.885741000	-0.328767000	-3.274366000
6	5.637399000	-1.472569000	-0.401444000
7	6.618401000	-1.055273000	0.461050000
6	6.612343000	-1.965406000	1.400531000
7	4.209933000	-1.597367000	-2.248566000
6	3.687320000	-2.765220000	-1.726690000
1	2.883962000	-3.202189000	-2.299315000
7	4.076596000	-3.343921000	-0.631958000
6	5.048072000	-2.669257000	0.010437000
6	5.194465000	-0.902810000	-1.596401000
7	5.721904000	0.215283000	-2.100314000
1	5.216122000	1.096611000	-2.046234000
1	6.714428000	0.345607000	-1.967592000
6	-2.963453000	-2.348753000	0.379503000
1	-3.936608000	-2.449910000	-0.096805000
1	-2.417731000	-3.291422000	0.301254000

1	-3.091240000	-2.131133000	1.441688000
6	-2.120945000	2.957159000	4.023169000
1	-1.460995000	2.950261000	4.893998000
1	-2.602127000	3.936167000	3.963221000
1	-2.897027000	2.210292000	4.180121000
6	2.238363000	3.379811000	-2.637367000
1	2.970040000	3.361743000	-3.444327000
1	1.393901000	3.995595000	-2.956808000
1	2.677471000	3.830385000	-1.748654000
6	-0.329625000	0.551677000	-8.375099000
1	0.559798000	0.092135000	-8.813141000
1	-1.172056000	0.336796000	-9.026472000
1	-0.195817000	1.634427000	-8.338483000
6	4.643645000	0.347178000	3.302134000
1	4.356767000	1.037238000	4.099747000
1	5.132433000	-0.519344000	3.750802000
1	5.373181000	0.834004000	2.658578000
1	5.534736000	-3.792086000	1.734445000
1	7.271288000	-1.988850000	2.249652000

^{5}R′

6	-0.180485000	2.097988000	-5.401748000
1	-0.514600000	1.540699000	-6.279931000
1	-0.895859000	2.904215000	-5.250340000
7	0.486419000	-0.025054000	-4.170297000
1	0.876270000	-0.476244000	-4.978783000
6	-0.131138000	1.229019000	-4.175777000
6	0.474970000	-0.526822000	-2.907681000
1	0.883353000	-1.472439000	-2.607113000
7	-0.146036000	0.330098000	-2.098891000
6	-0.530848000	1.427500000	-2.877107000
1	-1.058399000	2.253396000	-2.442400000
6	-1.788495000	3.978062000	1.463903000
1	-2.852580000	4.200346000	1.548801000
1	-1.306614000	4.299485000	2.390565000
6	-1.572144000	2.488868000	1.289812000
8	-0.613514000	2.077467000	0.496767000
8	-2.234436000	1.693161000	2.021640000
6	5.447613000	1.358328000	-0.957164000
1	6.048935000	1.993170000	-0.300102000
1	5.788935000	0.333069000	-0.820895000
7	3.279019000	2.652728000	-0.716410000
1	3.687382000	3.547422000	-0.922132000
6	3.982962000	1.449936000	-0.650794000
6	1.967404000	2.424835000	-0.473971000
1	1.187391000	3.157938000	-0.437070000
7	1.788394000	1.124555000	-0.226465000
6	3.036509000	0.506561000	-0.340006000
1	3.115084000	-0.550268000	-0.176342000
6	-5.009749000	0.328954000	6.305383000
1	-6.042267000	0.331085000	5.949003000
1	-4.982678000	-0.180587000	7.264466000
7	-4.105165000	-0.390024000	5.380116000
1	-3.745671000	-1.277554000	5.687433000
6	-3.697373000	0.062696000	4.173079000
7	-4.279064000	1.163621000	3.650159000
1	-5.093559000	1.580099000	4.058306000
1	-3.735433000	1.641436000	2.913570000
7	-2.715320000	-0.531353000	3.486536000
1	-2.130579000	-1.273710000	3.839277000
1	-2.381755000	-0.134468000	2.593529000
26	-0.035274000	0.278623000	-0.012402000
8	-1.512397000	-0.425250000	0.300448000
6	1.150340000	-3.590235000	1.085270000
1	0.686464000	-3.974771000	0.174697000
1	2.223206000	-3.517824000	0.904454000
6	0.580409000	-2.219978000	1.399034000
8	-0.110024000	-1.972033000	2.400211000
8	0.917673000	-1.280216000	0.491497000
7	-3.494814000	-0.339819000	-5.373648000
6	-4.552775000	0.683803000	-0.083063000
1	-3.602662000	0.656377000	0.449761000
1	-5.231504000	-0.058310000	0.338310000
1	-5.013878000	1.664663000	-0.038884000
6	-3.967648000	1.025113000	-3.732519000
7	-3.886061000	1.822847000	-4.863305000
6	-3.573578000	0.974468000	-5.831499000
1	-3.450264000	1.222273000	-6.869574000
7	-4.299288000	0.366047000	-1.507834000
6	-4.033722000	-0.947977000	-1.888764000
1	-4.020041000	-1.667571000	-1.087820000
7	-3.765341000	-1.333163000	-3.115492000
6	-3.725760000	-0.317871000	-4.021871000
6	-4.285174000	1.392400000	-2.424915000
7	-4.562736000	2.658699000	-2.098218000
1	-3.859508000	3.234108000	-1.635425000
1	-5.074988000	3.189679000	-2.787892000
1	5.667180000	1.644719000	-1.989898000
1	0.795520000	2.537553000	-5.628850000
1	0.960442000	-4.282214000	1.904055000
1	-4.672973000	1.355634000	6.451951000
1	-1.363387000	4.544658000	0.637754000
1	-3.288350000	-1.159554000	-5.916318000

^{5}TS$_{H,\pi}$

7	0.376268000	-0.034721000	-3.989944000
1	0.811889000	-0.511900000	-4.761532000
6	-0.189432000	1.230228000	-4.040606000
6	0.304679000	-0.501140000	-2.727451000
1	0.692382000	-1.447046000	-2.399934000
7	-0.306297000	0.380650000	-1.954714000
6	-0.615231000	1.462358000	-2.762723000
1	-1.081632000	2.341697000	-2.361729000
6	-1.426951000	2.722963000	1.541134000
8	-0.728055000	2.262255000	0.528012000
8	-1.791650000	2.020797000	2.481055000
7	3.263786000	2.632119000	-0.634063000
1	3.713256000	3.509483000	-0.835905000
6	3.890145000	1.396844000	-0.624051000
6	1.964857000	2.475980000	-0.330911000
1	1.244431000	3.266218000	-0.265142000
7	1.713782000	1.199255000	-0.076353000
6	2.905035000	0.516164000	-0.269257000
1	2.957382000	-0.544080000	-0.124641000
7	-4.009089000	-0.312073000	5.262949000
1	-3.648120000	-1.182584000	5.619928000
6	-3.636145000	0.049416000	4.022538000
7	-4.242136000	1.089711000	3.420944000
1	-5.044529000	1.519387000	3.846405000
1	-3.623222000	1.680434000	2.872296000
7	-2.676116000	-0.594156000	3.374957000
1	-2.038357000	-1.233642000	3.825319000
1	-2.365898000	-0.314710000	2.441750000
26	-0.199496000	0.456106000	0.145856000
8	-1.843437000	-0.185040000	0.626376000
6	0.464160000	-2.095795000	1.446282000
8	-0.061951000	-1.838377000	2.509335000
8	0.632714000	-1.213324000	0.468928000
7	-3.740867000	-0.364598000	-5.574671000
6	-4.243795000	0.399568000	-0.237264000
1	-4.625049000	-0.458570000	0.300675000
1	-4.662908000	1.334934000	0.104696000
1	-2.813751000	0.288050000	0.201527000
6	-4.030880000	0.957271000	-3.868420000
7	-3.926410000	1.786720000	-4.957726000
6	-3.719856000	0.965595000	-5.955257000
1	-3.599565000	1.260468000	-6.981732000
7	-4.257902000	0.204263000	-1.661373000

6	-4.167418000	-1.093464000	-2.145548000
1	-4.230228000	-1.859870000	-1.391072000
7	-4.006916000	-1.436908000	-3.386722000
6	-3.916302000	-0.385540000	-4.220564000
6	-4.196197000	1.273578000	-2.520014000
7	-4.312689000	2.535086000	-2.094977000
1	-4.314536000	3.265765000	-2.791145000
1	-4.445610000	2.805773000	-1.138949000
6	5.385537000	1.216490000	-0.944794000
1	5.617516000	1.565948000	-1.951877000
1	6.007115000	1.763393000	-0.234644000
1	5.648144000	0.163119000	-0.879901000
6	1.017753000	-3.487795000	1.089253000
1	2.068742000	-3.417862000	0.810906000
1	0.909880000	-4.149010000	1.943697000
1	0.468786000	-3.893709000	0.239038000
6	-1.678906000	4.240357000	1.616190000
1	-2.592598000	4.431290000	2.173091000
1	-0.853220000	4.695088000	2.167054000
1	-1.725001000	4.704199000	0.632628000
6	-0.225885000	2.096671000	-5.313221000
1	0.760942000	2.504566000	-5.537067000
1	-0.557053000	1.518938000	-6.176917000
1	-0.916402000	2.924963000	-5.173869000
6	-4.937845000	0.421192000	6.135092000
1	-5.973661000	0.327662000	5.803704000
1	-4.866672000	0.000865000	7.133597000
1	-4.662081000	1.473680000	6.197610000
1	-3.659873000	-1.170155000	-6.174721000

$^{5}TS_{H,\sigma}$

7	-0.149364000	-0.014035000	-4.166693000
1	-0.008489000	-0.514586000	-5.028458000
6	-0.306190000	1.371451000	-4.053917000
6	-0.120359000	-0.567563000	-2.924395000
1	0.007107000	-1.614071000	-2.722296000
7	-0.267463000	0.394764000	-2.011740000
6	-0.372941000	1.604031000	-2.704357000
1	-0.493683000	2.536298000	-2.189060000
6	-1.768654000	2.813656000	1.078394000
8	-0.658211000	2.204855000	0.780690000
8	-2.900368000	2.209995000	1.104520000
7	3.311169000	2.642831000	-0.641227000
1	3.772102000	3.514381000	-0.843082000
6	3.935908000	1.396301000	-0.602361000
6	1.995131000	2.495037000	-0.348525000
1	1.290811000	3.297155000	-0.262312000
7	1.739667000	1.208866000	-0.086059000
6	2.944095000	0.519353000	-0.246311000
1	2.992009000	-0.539458000	-0.091802000
7	-4.075084000	-0.173242000	5.118561000
1	-3.699106000	-1.028232000	5.495757000
6	-3.654978000	0.196482000	3.888517000
7	-4.236246000	1.239883000	3.263168000
1	-5.042277000	1.687323000	3.662232000
1	-3.782755000	1.681540000	2.460338000
7	-2.648175000	-0.451343000	3.289113000
1	-2.091476000	-1.153712000	3.752798000
1	-2.360269000	-0.288426000	2.320603000
26	-0.178774000	0.355372000	0.088787000
8	-1.840836000	-0.308940000	0.517027000
6	0.612882000	-2.249266000	1.523507000
8	-0.180999000	-2.280040000	2.474974000
8	0.701297000	-1.202759000	0.676296000
7	-3.745807000	-0.243514000	-5.438906000
6	-4.239412000	-0.043071000	-0.052601000
1	-4.572552000	-0.978060000	0.377765000
1	-4.689273000	0.835994000	0.379392000
1	-2.921066000	-0.007471000	0.305233000
6	-3.985058000	0.915437000	-3.594315000
7	-3.830459000	1.850116000	-4.610407000
6	-3.665665000	1.122019000	-5.708405000
1	-3.543464000	1.511046000	-6.702023000
7	-4.230824000	-0.075228000	-1.478738000
6	-4.213715000	-1.332461000	-2.092819000
1	-4.322636000	-2.171325000	-1.426667000
7	-4.069747000	-1.533604000	-3.373660000
6	-3.942421000	-0.385397000	-4.096030000
6	-4.085652000	1.102179000	-2.206723000
7	-4.043546000	2.294939000	-1.614068000
1	-4.031836000	3.111921000	-2.206161000
1	-3.873656000	2.415134000	-0.615210000
6	-1.711640000	4.309142000	1.441489000
1	-1.176501000	4.428878000	2.385101000
1	-1.160839000	4.868279000	0.685309000
1	-2.716104000	4.706906000	1.543886000
6	5.425366000	1.184715000	-0.931508000
1	5.647923000	1.482608000	-1.957161000
1	6.065249000	1.755865000	-0.257581000
1	5.673756000	0.131735000	-0.820314000
6	-0.350302000	2.297968000	-5.283228000
1	0.643534000	2.680357000	-5.519152000
1	-0.721630000	1.764535000	-6.157492000
1	-1.011290000	3.140662000	-5.094373000
6	1.604836000	-3.379841000	1.192712000
1	2.618707000	-3.055243000	1.431576000
1	1.363611000	-4.256750000	1.785694000
1	1.564717000	-3.618904000	0.131266000
6	-5.028397000	0.560501000	5.963332000
1	-6.049137000	0.489018000	5.583641000
1	-5.009035000	0.120843000	6.955690000
1	-4.741405000	1.608138000	6.056703000
1	-3.725978000	-0.995771000	-6.109768000

$^{5}I_{\pi}$

6	-0.180583000	2.209271000	-5.393963000
1	-0.507129000	1.669876000	-6.286507000
1	-0.891689000	3.016042000	-5.226191000
7	0.471424000	0.057176000	-4.198511000
1	0.874930000	-0.377790000	-5.009259000
6	-0.134842000	1.317916000	-4.186203000
6	0.445489000	-0.469082000	-2.949898000
1	0.839128000	-1.424983000	-2.661689000
7	-0.175123000	0.379632000	-2.128469000
6	-0.540377000	1.496436000	-2.889181000
1	-1.058915000	2.323378000	-2.445161000
6	-1.872620000	4.168060000	1.346548000
1	-2.438840000	4.263280000	2.274072000
1	-0.871001000	4.567703000	1.490781000
6	-1.830857000	2.725590000	0.915318000
8	-0.708016000	2.181912000	0.583856000
8	-2.946298000	2.057061000	0.913471000
6	5.437745000	1.325219000	-0.894946000
1	6.068367000	1.884591000	-0.198025000
1	5.750930000	0.282823000	-0.852171000
7	3.317602000	2.660048000	-0.503520000
1	3.754829000	3.553799000	-0.644674000
6	3.980557000	1.433258000	-0.552807000
6	2.003183000	2.457227000	-0.244544000
1	1.251622000	3.212332000	-0.132941000
7	1.780499000	1.147192000	-0.095869000
6	3.007966000	0.501204000	-0.290996000
1	3.062199000	-0.567843000	-0.219820000
6	-4.866626000	0.494626000	6.060765000
1	-5.898313000	0.330297000	5.740642000
1	-4.744556000	0.087160000	7.060464000
7	-3.893441000	-0.180455000	5.169234000
1	-3.505124000	-1.045382000	5.505216000
6	-3.546184000	0.179313000	3.908913000

7	-4.155877000	1.227504000	3.314322000
1	-5.009879000	1.610565000	3.675153000
1	-3.798497000	1.567283000	2.405563000
7	-2.592644000	-0.493058000	3.251233000
1	-1.952411000	-1.135070000	3.697168000
1	-2.344396000	-0.361942000	2.215214000
26	-0.129384000	0.262957000	-0.057706000
8	-1.861158000	-0.280864000	0.482155000
6	1.060449000	-3.670035000	1.073797000
1	0.528581000	-4.089909000	0.218594000
1	2.118800000	-3.624280000	0.811672000
6	0.538688000	-2.279808000	1.360216000
8	0.027407000	-1.936015000	2.439215000
8	0.683060000	-1.413896000	0.336079000
7	-3.537031000	-0.259410000	-5.246758000
6	-4.959335000	0.177725000	-0.049247000
1	-4.937350000	-0.714326000	0.547305000
1	-5.091073000	1.144777000	0.379124000
1	-2.600282000	0.328007000	0.309302000
6	-4.026592000	0.964556000	-3.494074000
7	-3.816368000	1.861945000	-4.535296000
6	-3.508955000	1.093912000	-5.574029000
1	-3.301364000	1.442701000	-6.568713000
7	-4.524202000	0.068543000	-1.365340000
6	-4.293317000	-1.220118000	-1.897837000
1	-4.406865000	-2.021595000	-1.191657000
7	-3.973994000	-1.462290000	-3.138811000
6	-3.853122000	-0.353547000	-3.919343000
6	-4.314546000	1.210264000	-2.147055000
7	-4.391667000	2.429209000	-1.605532000
1	-4.302024000	3.209556000	-2.232845000
1	-4.091171000	2.549307000	-0.640150000
1	5.648667000	1.692210000	-1.903769000
1	0.795785000	2.652755000	-5.610536000
1	-3.367767000	-1.035980000	-5.860054000
1	-2.387160000	4.769292000	0.591942000
1	-4.666791000	1.565778000	6.102773000
1	0.927746000	-4.318481000	1.938155000

^{5}TS$_{reb}$

7	0.090483000	-0.251137000	-3.994470000
1	0.393035000	-0.795005000	-4.785335000
6	-0.335947000	1.074652000	-4.042259000
6	0.120462000	-0.658310000	-2.701567000
1	0.437773000	-1.626965000	-2.364396000
7	-0.305894000	0.330103000	-1.918572000
6	-0.607113000	1.418121000	-2.740496000
1	-0.938712000	2.358632000	-2.347457000
6	-1.611082000	2.708569000	1.274141000
8	-0.525928000	2.132118000	0.890267000
8	-2.724530000	2.037727000	1.319536000
7	3.123745000	2.702731000	-0.726208000
1	3.475699000	3.632891000	-0.877593000
6	3.839383000	1.535073000	-0.966503000
6	1.881864000	2.389189000	-0.305730000
1	1.128245000	3.094765000	-0.030366000
7	1.766664000	1.062108000	-0.208547000
6	3.004696000	0.511327000	-0.566298000
1	3.164835000	-0.548804000	-0.561153000
7	-4.368837000	-0.125776000	5.248480000
1	-4.048084000	-0.978685000	5.679429000
6	-3.823678000	0.205300000	4.055605000
7	-4.342639000	1.242105000	3.369649000
1	-5.190780000	1.682304000	3.679331000
1	-3.811686000	1.682498000	2.616154000
7	-2.783945000	-0.472569000	3.545022000
1	-2.224436000	-1.113023000	4.087663000
1	-2.460476000	-0.362022000	2.592554000
26	-0.064733000	0.261635000	0.150558000
8	-1.862496000	-0.257934000	0.479107000
6	0.553713000	-2.248526000	1.702897000
8	0.057314000	-1.906034000	2.794303000
8	0.712805000	-1.396489000	0.679053000
7	-3.456125000	0.212335000	-5.558281000
6	-4.010650000	-0.255205000	-0.179533000
1	-4.152199000	-1.256022000	0.183890000
1	-4.332908000	0.578273000	0.415848000
1	-2.237431000	0.568654000	0.887019000
6	-3.728130000	1.132727000	-3.575074000
7	-3.545793000	2.186276000	-4.467013000
6	-3.356723000	1.599803000	-5.644690000
1	-3.212067000	2.112785000	-6.576663000
7	-3.990656000	-0.104227000	-1.569050000
6	-3.986023000	-1.277030000	-2.357282000
1	-4.113434000	-2.193593000	-1.806908000
7	-3.829825000	-1.311757000	-3.652232000
6	-3.670423000	-0.093380000	-4.237852000
6	-3.864197000	1.153380000	-2.178013000
7	-3.872429000	2.286257000	-1.470695000
1	-3.887792000	3.154984000	-1.983770000
1	-3.744137000	2.323946000	-0.464527000
6	5.207054000	1.620191000	-1.669234000
1	5.085274000	1.997499000	-2.685812000
1	5.890937000	2.277939000	-1.131413000
1	5.666395000	0.635669000	-1.723728000
6	1.091322000	-3.665125000	1.427536000
1	2.166125000	-3.620875000	1.250254000
1	0.890796000	-4.300424000	2.284526000
1	0.619912000	-4.078219000	0.535927000
6	-1.605110000	4.199069000	1.661405000
1	-1.272423000	4.801140000	0.814690000
1	-2.597269000	4.513039000	1.968343000
1	-0.897836000	4.360950000	2.475084000
6	-5.446916000	0.594912000	5.940761000
1	-6.404439000	0.482103000	5.429169000
1	-5.546246000	0.176223000	6.937237000
1	-5.204757000	1.652360000	6.047646000
6	-0.325862000	1.869486000	-5.361250000
1	0.698405000	2.140539000	-5.625908000
1	-0.735960000	1.287086000	-6.184728000
1	-0.897575000	2.785886000	-5.252149000
1	-3.423957000	-0.450105000	-6.317158000

^{5}P

6	-0.052230000	2.224296000	-5.370916000
1	-0.367474000	1.658195000	-6.250577000
1	-0.798900000	2.998018000	-5.201325000
7	0.674618000	0.096795000	-4.178300000
1	1.051490000	-0.341098000	-4.999974000
6	0.050334000	1.347545000	-4.151979000
6	0.707923000	-0.411983000	-2.916727000
1	1.119542000	-1.366170000	-2.649267000
7	0.107911000	0.429239000	-2.076040000
6	-0.308999000	1.528230000	-2.838404000
1	-0.846633000	2.336324000	-2.383066000
6	-1.743438000	4.041119000	1.306645000
1	-1.681257000	4.137968000	2.394873000
1	-0.843638000	4.462926000	0.865966000
6	-1.890795000	2.581903000	0.937007000
8	-0.962272000	1.965147000	0.308765000
8	-2.987098000	1.974007000	1.287217000
6	5.806371000	1.638028000	-1.178145000
1	6.437520000	2.328258000	-0.610522000
1	6.184971000	0.631618000	-1.003022000
7	3.627504000	2.896154000	-0.847135000
1	3.992827000	3.783353000	-1.142968000
6	4.367619000	1.718419000	-0.771485000
6	2.338365000	2.641934000	-0.509926000

1	1.551992000	3.369393000	-0.478341000
7	2.216465000	1.353075000	-0.192781000
6	3.471858000	0.767077000	-0.353809000
1	3.628532000	-0.277716000	-0.162906000
6	-4.731187000	0.539405000	6.484741000
1	-5.800416000	0.389187000	6.318698000
1	-4.479125000	0.178702000	7.478001000
7	-3.909902000	-0.201368000	5.501587000
1	-3.558219000	-1.099399000	5.789809000
6	-3.737683000	0.112725000	4.199471000
7	-4.365106000	1.180867000	3.661902000
1	-5.138188000	1.628265000	4.118647000
1	-3.988900000	1.584008000	2.790697000
7	-2.938019000	-0.614924000	3.411134000
1	-2.172628000	-1.221171000	3.681803000
1	-3.102664000	-0.599188000	2.402281000
26	0.383458000	0.435653000	0.049118000
8	-3.815062000	-0.302789000	0.713022000
6	0.697461000	-3.712857000	1.117955000
1	0.462066000	-4.421358000	1.909998000
1	0.364771000	-4.115462000	0.159774000
6	0.017930000	-2.374726000	1.374010000
8	-0.749653000	-2.185153000	2.342968000
8	0.291178000	-1.438565000	0.462108000
7	-3.452663000	-0.278459000	-5.362068000
6	-4.944067000	-0.065555000	-0.080116000
1	-5.674880000	-0.842458000	0.140096000
1	-5.384246000	0.914561000	0.103897000
1	-3.273774000	0.551943000	0.795458000
6	-3.943396000	0.885112000	-3.572027000
7	-3.627923000	1.817029000	-4.552432000
6	-3.318007000	1.083434000	-5.614828000
1	-3.039771000	1.462099000	-6.580337000
7	-4.655288000	-0.123814000	-1.516672000
6	-4.484715000	-1.343320000	-2.133956000
1	-4.701206000	-2.199455000	-1.511305000
7	-4.123694000	-1.556528000	-3.373251000
6	-3.845561000	-0.417734000	-4.062599000
6	-4.276363000	1.073119000	-2.222640000
7	-4.300849000	2.285372000	-1.654211000
1	-4.431752000	3.073019000	-2.265547000
1	-3.811004000	2.474483000	-0.789367000
1	-4.503053000	1.604383000	6.443538000
1	0.900587000	2.708878000	-5.606036000
1	-3.296403000	-1.033080000	-6.006207000
1	5.943299000	1.861485000	-2.240417000
1	1.777993000	-3.573508000	1.057282000
1	-2.614313000	4.614435000	0.982477000

DFT Model calculations:

$^5R_{Cyt}$:

26	-0.738662000	0.464234000	1.032331000
7	-0.321216000	-0.245227000	2.948123000
6	0.872225000	-0.110458000	3.536506000
7	0.791986000	-0.601469000	4.806648000
6	-0.507925000	-1.058697000	5.041531000
6	-1.184764000	-0.827015000	3.867030000
1	1.726912000	0.362070000	3.076786000
1	1.545141000	-0.614674000	5.473087000
1	-2.214591000	-0.994652000	3.620648000
7	-0.838746000	-1.452746000	0.350026000
6	-1.752044000	-2.378146000	0.654232000
7	-1.418752000	-3.549623000	0.049061000
6	-0.243798000	-3.366229000	-0.690384000
6	0.101886000	-2.054439000	-0.493334000
1	-2.610397000	-2.222597000	1.277921000
1	-1.938494000	-4.408126000	0.115781000
1	0.924937000	-1.489211000	-0.878778000
8	-2.326961000	1.107305000	-0.425508000
6	-3.250161000	0.601467000	0.339693000
8	-2.879220000	0.044741000	1.444338000
6	-4.690552000	0.629733000	-0.065403000
1	-5.331020000	0.686574000	0.812754000
1	-4.936192000	-0.288489000	-0.605041000
1	-4.890149000	1.468082000	-0.729610000
6	2.038130000	1.263920000	0.327540000
8	0.900946000	0.585515000	0.056860000
6	2.751509000	1.770265000	-0.900945000
1	3.076303000	0.930368000	-1.520148000
1	3.619180000	2.355659000	-0.610837000
1	2.076371000	2.375660000	-1.509268000
8	-0.645209000	2.027894000	1.578349000
8	2.479938000	1.415772000	1.480817000
7	-1.487946000	5.821050000	1.399149000
6	-1.494744000	7.256597000	1.597680000
6	-1.818977000	5.244346000	0.211043000
6	-1.906194000	8.044637000	0.449061000
7	-2.206157000	6.060534000	-0.818856000
6	-2.252422000	7.448957000	-0.703226000
1	-1.927127000	9.113394000	0.576568000
1	-2.429209000	5.638958000	-1.705039000
1	-2.577221000	7.976112000	-1.585830000
6	-1.158616000	4.983314000	2.582883000
1	-2.078907000	4.599260000	3.021946000
1	-0.518728000	4.144218000	2.317352000
6	0.390388000	-4.455658000	-1.486567000
1	0.666278000	-5.309593000	-0.863010000
1	-0.269679000	-4.818340000	-2.278525000
1	1.299297000	-4.088871000	-1.960292000
6	-0.934145000	-1.656908000	6.341789000
1	-1.995160000	-1.899464000	6.312398000
1	-0.391009000	-2.580158000	6.564668000
1	-0.779766000	-0.968509000	7.177367000
1	-0.659462000	5.627621000	3.296420000
8	-1.177280000	7.722824000	2.694276000
7	-1.791773000	3.937716000	0.045093000
1	-2.078685000	3.469708000	-0.806819000
1	-1.404164000	3.266466000	0.739990000

$^5TS_{H,Cyt}$:

26	-0.474112000	0.199991000	0.087696000
7	0.051741000	0.575262000	2.133660000
6	1.278766000	0.593328000	2.657989000
7	1.191647000	0.791261000	4.003954000
6	-0.156306000	0.908755000	4.363050000
6	-0.849029000	0.768882000	3.186697000
1	2.193221000	0.481944000	2.109735000
1	1.969320000	0.847031000	4.639597000
1	-1.904848000	0.792186000	3.004795000
7	-0.411579000	-1.851040000	0.362423000
6	0.577217000	-2.661996000	-0.017812000
7	0.229276000	-3.947825000	0.262642000
6	-1.042294000	-3.963937000	0.847783000
6	-1.424907000	-2.647406000	0.902966000
1	1.491299000	-2.359178000	-0.491505000
1	0.788940000	-4.760756000	0.067659000
1	-2.336309000	-2.213993000	1.266412000
8	-0.816392000	0.068633000	-1.842746000
6	-1.865983000	0.268860000	-2.626221000
8	-2.558621000	1.324846000	-2.583657000
6	-2.175965000	-0.819581000	-3.614482000
1	-3.116377000	-0.614336000	-4.116474000
1	-2.222538000	-1.784129000	-3.109226000
1	-1.378167000	-0.879092000	-4.357335000
6	1.983543000	1.791322000	-0.979108000
8	1.357856000	0.841974000	-0.314253000
6	3.272733000	1.399865000	-1.649360000

1	3.816838000	0.667822000	-1.055851000
1	3.887748000	2.276758000	-1.829203000
1	3.043207000	0.937581000	-2.613007000
8	-2.153933000	0.650962000	0.484551000
8	1.540345000	2.976959000	-1.080282000
7	-2.469577000	3.660914000	0.676685000
6	-2.576956000	4.306359000	1.980835000
6	-1.339580000	3.772691000	-0.102065000
6	-1.438406000	5.104365000	2.367913000
7	-0.266687000	4.449299000	0.407229000
6	-0.323002000	5.127299000	1.607848000
1	-1.505934000	5.640155000	3.298724000
1	0.617741000	4.273335000	-0.077134000
1	0.572298000	5.662245000	1.881604000
6	-3.509939000	2.741102000	0.306366000
1	-3.790166000	2.771263000	-0.741238000
1	-3.001288000	1.571594000	0.436237000
6	-1.716395000	-5.219788000	1.286338000
1	-2.732835000	-5.004004000	1.610006000
1	-1.778374000	-5.951929000	0.478428000
1	-1.196499000	-5.689404000	2.124738000
6	-0.605669000	1.120458000	5.769609000
1	-1.667888000	1.356954000	5.791276000
1	-0.453512000	0.230032000	6.384427000
1	-0.075993000	1.948359000	6.245935000
1	-4.330510000	2.803958000	1.009002000
8	-3.595508000	4.119137000	2.659887000
7	-1.240429000	3.235915000	-1.300266000
1	-0.319346000	3.267228000	-1.730672000
1	-1.910183000	2.559492000	-1.743072000

^{5}R$_{Thy}$:

26	-0.646585000	0.767026000	1.172589000
7	-0.437834000	-0.282915000	2.937939000
6	0.721327000	-0.458933000	3.570305000
7	0.477666000	-1.089469000	4.756180000
6	-0.898674000	-1.314698000	4.885830000
6	-1.453637000	-0.802735000	3.740311000
1	1.677110000	-0.102456000	3.210965000
1	1.177858000	-1.331402000	5.434987000
1	-2.480654000	-0.729309000	3.439452000
7	-0.787615000	-1.013552000	0.138383000
6	-1.797165000	-1.883255000	0.146967000
7	-1.489468000	-2.937413000	-0.662759000
6	-0.220368000	-2.728969000	-1.216383000
6	0.199036000	-1.528394000	-0.704169000
1	-2.712307000	-1.762233000	0.691885000
1	-2.080761000	-3.730833000	-0.837375000
1	1.109423000	-0.985195000	-0.856141000
8	-1.972779000	1.594244000	-0.213460000
6	-3.079740000	1.204671000	0.354608000
8	-3.001437000	0.453371000	1.394635000
6	-4.392746000	1.662735000	-0.202541000
1	-5.206065000	1.066295000	0.202036000
1	-4.384986000	1.607234000	-1.289967000
1	-4.555558000	2.707763000	0.069541000
6	2.288960000	1.427560000	0.956031000
8	1.167082000	0.847108000	0.506218000
6	2.900934000	2.434433000	0.016457000
1	2.996103000	2.012444000	-0.984224000
1	3.873393000	2.750560000	0.383332000
1	2.241918000	3.301482000	-0.061482000
8	-0.591314000	2.202441000	1.988075000
8	2.805692000	1.139111000	2.056549000
7	-1.085715000	5.943850000	1.712104000
6	-1.263987000	5.147398000	0.586988000
6	-1.191476000	7.330996000	1.649609000
6	-1.545537000	5.646646000	-0.645101000
7	-1.480346000	7.821981000	0.383271000
6	-1.671711000	7.087533000	-0.801440000
1	-1.561425000	8.825677000	0.308267000
6	-0.779997000	5.350970000	3.027328000
1	-1.575405000	5.581532000	3.734637000
1	-0.685590000	4.275906000	2.907554000
6	0.421079000	-3.691748000	-2.157938000
1	0.553912000	-4.679156000	-1.707808000
1	-0.160855000	-3.817940000	-3.074753000
1	1.406397000	-3.327850000	-2.444254000
6	-1.502727000	-1.973134000	6.080598000
1	-2.584571000	-2.026213000	5.968435000
1	-1.135065000	-2.993722000	6.217816000
1	-1.296043000	-1.418686000	6.999807000
1	0.148765000	5.766820000	3.413332000
1	-1.159684000	4.086192000	0.763278000
8	-1.040993000	8.070100000	2.643822000
8	-1.929784000	7.683657000	-1.870867000
6	-1.728595000	4.780257000	-1.859636000
1	-1.682684000	3.724177000	-1.596963000
1	-2.689145000	4.984644000	-2.338186000
1	-0.961837000	4.991896000	-2.609212000

^{5}TS$_{H,Thy}$:

26	-0.931710000	1.347308000	1.733193000
7	-0.454439000	0.178794000	3.406336000
6	0.770231000	0.043187000	3.915256000
7	0.685783000	-0.676911000	5.071685000
6	-0.654469000	-1.008703000	5.312101000
6	-1.349473000	-0.465393000	4.262195000
1	1.665314000	0.476656000	3.487685000
1	1.463198000	-0.910650000	5.663602000
1	-2.402112000	-0.458756000	4.055197000
7	-0.944288000	-0.411319000	0.436935000
6	-1.815378000	-1.417737000	0.438253000
7	-1.439460000	-2.351294000	-0.487741000
6	-0.265472000	-1.914770000	-1.113402000
6	0.024326000	-0.710751000	-0.523271000
1	-2.682758000	-1.481496000	1.065285000
1	-1.924114000	-3.208504000	-0.686727000
1	0.839174000	-0.035918000	-0.691468000
8	-2.578789000	2.107838000	0.410052000
6	-3.483795000	1.450347000	1.065154000
8	-3.115399000	0.667848000	2.024025000
6	-4.936679000	1.627007000	0.729495000
1	-5.515801000	0.776679000	1.081523000
1	-5.065907000	1.756274000	-0.343477000
1	-5.318736000	2.524761000	1.221892000
6	2.004604000	2.177993000	1.337214000
8	0.834280000	1.653136000	0.970270000
6	2.494996000	3.312266000	0.471046000
1	2.453519000	3.033908000	-0.582229000
1	3.509995000	3.584892000	0.746285000
1	1.840246000	4.175865000	0.602898000
8	-1.045782000	2.840189000	2.679294000
8	2.668029000	1.757530000	2.312159000
7	-1.493773000	5.728400000	1.302800000
6	-1.636259000	5.118933000	0.055130000
6	-0.727393000	6.899474000	1.465522000
6	-1.094279000	5.619407000	-1.082137000
7	-0.183620000	7.381804000	0.286550000
6	-0.311759000	6.845868000	-1.008523000
1	0.362647000	8.227480000	0.369523000
6	-2.064728000	5.116857000	2.465302000
1	-3.094150000	4.807304000	2.308252000
1	-1.487093000	4.014744000	2.674942000
6	0.421690000	-2.695487000	-2.183075000
1	0.746105000	-3.678512000	-1.830699000
1	-0.218676000	-2.848929000	-3.056011000
1	1.308463000	-2.160164000	-2.518723000

6	-1.093601000	-1.786654000	6.507100000
1	-2.175220000	-1.912709000	6.491188000
1	-0.646041000	-2.784063000	6.536825000
1	-0.837171000	-1.279561000	7.441242000
1	-1.909334000	5.731544000	3.342646000
1	-2.182242000	4.184356000	0.069716000
8	-0.563703000	7.459233000	2.563569000
8	0.233890000	7.416234000	-1.976049000
6	-1.249552000	4.956251000	-2.420426000
1	-1.803569000	4.022936000	-2.329340000
1	-1.771689000	5.609407000	-3.123425000
1	-0.274426000	4.740940000	-2.861710000

$^5R_{Gua}$:

7	1.719405000	1.841754000	-4.343992000
1	2.597671000	1.991367000	-4.813324000
6	0.462494000	1.866327000	-4.959896000
6	1.569836000	1.581571000	-3.020690000
1	2.376580000	1.498901000	-2.320034000
7	0.266330000	1.433001000	-2.749194000
6	-0.429337000	1.605921000	-3.951747000
1	-1.497129000	1.529305000	-3.985133000
6	-2.408961000	3.250312000	0.103438000
8	-1.242587000	2.694603000	-0.161164000
8	-3.436398000	2.584840000	0.442871000
7	2.688019000	2.502388000	1.122316000
1	3.184067000	3.248496000	1.581753000
6	3.134831000	1.178581000	1.020267000
6	1.475045000	2.617891000	0.523148000
1	0.888434000	3.511931000	0.462384000
7	1.116108000	1.426043000	0.031418000
6	2.142545000	0.522221000	0.336726000
1	2.060253000	-0.511204000	0.068321000
7	-3.857136000	-1.210655000	3.630532000
1	-3.483587000	-1.987966000	3.110568000
6	-3.874506000	-0.010369000	3.014876000
7	-4.378725000	1.066180000	3.659860000
1	-4.755804000	1.007575000	4.587943000
1	-4.385703000	1.965991000	3.211406000
7	-3.403183000	0.121510000	1.775586000
1	-2.997908000	-0.657064000	1.245439000
1	-3.415320000	1.021641000	1.288596000
26	-0.613189000	1.012911000	-0.943876000
8	-2.060142000	0.670513000	-1.687689000
6	-1.063378000	-1.872906000	-0.241278000
8	-2.254077000	-1.818777000	0.163926000
8	-0.295583000	-0.797681000	-0.402923000
7	-7.636534000	-2.580059000	-3.151730000
6	-8.484879000	-0.826755000	-2.114503000
7	-9.564671000	-1.586217000	-2.550454000
6	-9.038966000	-2.624887000	-3.165973000
1	-9.580500000	-3.425447000	-3.633209000
7	-7.117435000	0.895034000	-1.224683000
6	-5.955852000	0.278236000	-1.571261000
7	-6.019499000	-0.944739000	-2.185699000
6	-7.293586000	-1.433938000	-2.480529000
6	-8.486696000	0.422939000	-1.413044000
6	4.431580000	0.714994000	1.591923000
1	5.281474000	1.247502000	1.159648000
1	4.468697000	0.852006000	2.674777000
1	4.573131000	-0.345204000	1.391717000
6	-0.386570000	-3.180584000	-0.547890000
1	0.481866000	-3.313816000	0.098680000
1	-1.075626000	-4.005544000	-0.395967000
1	-0.024496000	-3.185616000	-1.576568000
6	-2.449851000	4.750961000	0.015193000
1	-3.452856000	5.116588000	0.211297000
1	-1.763406000	5.188284000	0.742261000
1	-2.124047000	5.078957000	-0.972194000
6	0.280176000	2.133571000	-6.415178000
1	0.659535000	3.118020000	-6.697058000
1	0.789944000	1.389711000	-7.031169000
1	-0.776533000	2.104554000	-6.673140000
6	-4.341488000	-1.463249000	4.994323000
1	-5.408614000	-1.254842000	5.086282000
1	-4.188960000	-2.513825000	5.220745000
1	-3.785682000	-0.880277000	5.730340000
1	-7.028824000	-3.250823000	-3.590038000
7	-4.784730000	0.850884000	-1.305438000
1	-4.695051000	1.679767000	-0.723849000
1	-3.875585000	0.499323000	-1.606184000
1	-7.073191000	1.813701000	-0.802787000
8	-9.406682000	1.115868000	-0.990546000
6	-4.790924000	-1.717886000	-2.476844000
1	-4.100647000	-1.659810000	-1.636923000
1	-5.057919000	-2.762386000	-2.602455000
1	-4.308316000	-1.355675000	-3.383557000

$^5TS_{H,Gua}$:

7	1.551189000	-1.239903000	-3.943242000
1	2.124399000	-1.940628000	-4.383696000
6	1.040168000	-0.103030000	-4.576751000
6	1.158160000	-1.243972000	-2.639796000
1	1.418010000	-1.995039000	-1.919485000
7	0.414393000	-0.160909000	-2.403764000
6	0.334322000	0.557955000	-3.601307000
1	-0.204037000	1.483751000	-3.659958000
6	-1.956070000	2.939897000	-0.499608000
8	-1.015879000	2.174770000	-1.037146000
8	-2.624817000	2.600472000	0.515129000
7	2.925535000	2.701297000	0.258345000
1	3.477371000	3.534466000	0.135494000
6	3.286755000	1.604821000	1.052374000
6	1.720734000	2.465835000	-0.316769000
1	1.201657000	3.133229000	-0.974360000
7	1.280814000	1.261212000	0.071072000
6	2.251381000	0.716192000	0.921464000
1	2.120675000	-0.255447000	1.349948000
7	-3.239995000	0.447119000	4.581733000
1	-3.010950000	-0.532847000	4.586424000
6	-3.160460000	1.099464000	3.403345000
7	-3.470437000	2.409620000	3.325265000
1	-3.800083000	2.931399000	4.116008000
1	-3.357685000	2.884046000	2.438408000
7	-2.784587000	0.464386000	2.288188000
1	-2.426083000	-0.490242000	2.243688000
1	-2.710152000	1.015089000	1.433665000
26	-0.400656000	0.391465000	-0.562616000
8	-2.036427000	-0.317323000	-0.722436000
6	-0.488455000	-2.125788000	1.106439000
8	-1.630218000	-1.994584000	1.622476000
8	0.148279000	-1.160230000	0.446504000
7	-6.623064000	1.217826000	-2.645067000
1	-2.856240000	0.005345000	-1.486373000
6	-7.562616000	-0.077684000	-1.127042000
7	-8.569676000	0.738091000	-1.624062000
6	-7.990074000	1.503544000	-2.526599000
1	-8.470638000	2.260297000	-3.117371000
7	-6.346010000	-1.731270000	0.064778000
6	-5.160650000	-1.412110000	-0.512682000
7	-5.146106000	-0.444536000	-1.507247000
6	-6.357772000	0.215803000	-1.746093000
6	-7.658105000	-1.124908000	-0.153165000
6	4.560303000	1.550775000	1.825496000
1	5.434138000	1.625177000	1.174656000
1	4.622666000	2.354883000	2.561867000
1	4.632817000	0.608000000	2.364032000
6	0.271987000	-3.422482000	1.212055000

1	-0.196684000	-4.072336000	1.944683000
1	0.275573000	-3.930099000	0.244586000
1	1.309664000	-3.242250000	1.488459000
6	-2.149594000	4.281736000	-1.154457000
1	-3.162272000	4.639125000	-0.985997000
1	-1.464801000	5.006627000	-0.707069000
1	-1.935049000	4.236851000	-2.219409000
6	1.294843000	0.207915000	-6.012912000
1	2.360747000	0.325574000	-6.219379000
1	0.912533000	-0.573765000	-6.672842000
1	0.804302000	1.139317000	-6.289122000
6	-3.627108000	1.066968000	5.856230000
1	-4.652507000	1.438856000	5.826186000
1	-3.571178000	0.311904000	6.634146000
1	-2.948488000	1.876858000	6.127777000
1	-5.990510000	1.665650000	-3.285386000
6	-3.964788000	-0.211486000	-2.299675000
1	-3.607691000	-1.104007000	-2.806479000
1	-4.060573000	0.654713000	-2.939610000
7	-4.023468000	-1.989648000	-0.137277000
1	-4.016999000	-2.719540000	0.558280000
1	-3.103367000	-1.535979000	-0.309099000
1	-6.362331000	-2.442136000	0.785648000
8	-8.610066000	-1.544667000	0.493646000

$^{5}R_{Ade}$:

6	0.870825000	2.592123000	-5.791586000
1	1.176342000	1.830963000	-6.512746000
1	-0.194614000	2.765627000	-5.929892000
7	2.430773000	1.871252000	-3.923267000
1	3.270997000	1.907895000	-4.476517000
6	1.144949000	2.168622000	-4.388531000
6	2.365855000	1.522630000	-2.611932000
1	3.208887000	1.238522000	-2.014086000
7	1.092263000	1.579411000	-2.205634000
6	0.325592000	1.978334000	-3.305519000
1	-0.736392000	2.089632000	-3.222331000
6	-0.565589000	5.027142000	1.227963000
1	-1.480804000	5.564307000	1.457970000
1	0.153827000	5.202371000	2.030782000
6	-0.842587000	3.552805000	1.101059000
8	0.160996000	2.832119000	0.621374000
8	-1.943032000	3.056959000	1.469619000
6	5.468980000	-0.322881000	1.584403000
1	5.627769000	-0.314143000	2.664947000
1	5.388728000	-1.363030000	1.274661000
7	4.060467000	1.788472000	1.464911000
1	4.728366000	2.381173000	1.929606000
6	4.236802000	0.424119000	1.200580000
6	2.839371000	2.180411000	1.016717000
1	2.427037000	3.165459000	1.098574000
7	2.216464000	1.130974000	0.468734000
6	3.078581000	0.031967000	0.578542000
1	2.776226000	-0.935366000	0.233166000
6	-4.122486000	-0.639840000	5.196897000
1	-5.080683000	-0.118519000	5.230979000
1	-4.299087000	-1.675386000	5.470764000
7	-3.540750000	-0.610872000	3.849724000
1	-3.377566000	-1.489120000	3.385616000
6	-3.180549000	0.511318000	3.190709000
7	-3.371125000	1.724436000	3.755358000
1	-3.780597000	1.837379000	4.663790000
1	-3.056369000	2.545411000	3.256427000
7	-2.643053000	0.450546000	1.972202000
1	-2.360011000	-0.412766000	1.498343000
1	-2.328332000	1.328572000	1.550924000
26	0.338862000	1.145516000	-0.330224000
8	-1.224017000	1.156927000	-0.897234000
6	0.029042000	-3.076232000	-0.480939000
1	0.428964000	-2.988897000	-1.490944000
1	0.840854000	-3.418847000	0.163107000
6	-0.479131000	-1.743025000	-0.001928000
8	-1.636469000	-1.605557000	0.462090000
8	0.410806000	-0.751049000	-0.083777000
7	-5.917936000	-2.730797000	-3.751514000
6	-4.392481000	1.879425000	-1.161989000
1	-3.343788000	1.805111000	-0.890963000
1	-4.984244000	2.019155000	-0.258488000
1	-4.518568000	2.728060000	-1.833814000
6	-6.344738000	-0.704512000	-3.018847000
7	-7.506997000	-1.131669000	-3.646088000
6	-7.218765000	-2.353326000	-4.076323000
1	-7.891033000	-2.995805000	-4.613722000
7	-4.798427000	0.628237000	-1.851326000
6	-3.870257000	-0.415337000	-1.948413000
1	-2.911620000	-0.225214000	-1.490320000
7	-4.103650000	-1.562753000	-2.545090000
6	-5.350336000	-1.682662000	-3.074538000
6	-6.069680000	0.513301000	-2.379376000
7	-6.974354000	1.496251000	-2.287200000
1	-6.807846000	2.373603000	-1.827617000
1	-7.881829000	1.343628000	-2.701858000
1	6.362355000	0.092743000	1.113153000
1	1.389216000	3.519450000	-6.044779000
1	-0.772728000	-3.807750000	-0.462382000
1	-3.444130000	-0.210393000	5.936010000
1	-0.128490000	5.413193000	0.308114000
1	-5.469087000	-3.606708000	-3.965905000

$^{5}TS_{H,Ade}$:

7	2.087352000	1.619551000	-4.204554000
1	2.720960000	1.287885000	-4.913036000
6	1.490337000	2.883810000	-4.185788000
6	1.682839000	0.915066000	-3.111402000
1	1.996146000	-0.081256000	-2.867308000
7	0.849851000	1.667177000	-2.389101000
6	0.723147000	2.895709000	-3.046333000
1	0.112610000	3.680647000	-2.644508000
6	-1.716498000	3.192448000	0.736434000
8	-0.731757000	2.865638000	-0.091579000
8	-2.347537000	2.342208000	1.421065000
7	3.138773000	2.816031000	1.494896000
1	3.645154000	3.615724000	1.837869000
6	3.534721000	1.481397000	1.654015000
6	1.969750000	2.861948000	0.809426000
1	1.435030000	3.751898000	0.545859000
7	1.587680000	1.612268000	0.515981000
6	2.557480000	0.745672000	1.035509000
1	2.468488000	-0.313396000	0.911173000
7	-2.973957000	-1.689560000	3.612488000
1	-2.746176000	-2.514791000	3.083313000
6	-2.894908000	-0.501840000	2.975356000
7	-3.239973000	0.637677000	3.609380000
1	-3.610250000	0.641776000	4.541404000
1	-3.114943000	1.518824000	3.126479000
7	-2.482784000	-0.430793000	1.707089000
1	-2.071712000	-1.200650000	1.177404000
1	-2.422708000	0.490416000	1.278190000
26	-0.018759000	1.121720000	-0.563210000
8	-1.608138000	0.530176000	-1.145534000
6	0.034993000	-1.886548000	-0.515109000
8	-1.113960000	-2.103565000	-0.054543000
8	0.626220000	-0.689358000	-0.519564000
7	-8.119627000	-1.072917000	-2.126404000
6	-3.300898000	1.433735000	-2.740176000
1	-3.391660000	2.508492000	-2.829575000
1	-2.768299000	0.948948000	-3.553543000
1	-2.349985000	1.178355000	-1.730053000

6	-5.922575000	-1.096804000	-2.060501000
7	-6.324825000	-2.397637000	-1.791924000
6	-7.651305000	-2.352915000	-1.847687000
1	-8.307215000	-3.190472000	-1.701019000
7	-4.565313000	0.800197000	-2.438391000
6	-5.746242000	1.555575000	-2.585981000
1	-5.599494000	2.601001000	-2.800333000
7	-6.957592000	1.074990000	-2.506928000
6	-7.022095000	-0.265534000	-2.263905000
6	-4.621162000	-0.557748000	-2.104632000
7	-3.524933000	-1.256370000	-1.840155000
1	-3.632901000	-2.238455000	-1.629434000
1	-2.622198000	-0.816041000	-1.605532000
6	4.782063000	1.087378000	2.369290000
1	5.672024000	1.506669000	1.895002000
1	4.773487000	1.414002000	3.411430000
1	4.889638000	0.004526000	2.367006000
6	0.867239000	-2.996481000	-1.103134000
1	0.423982000	-3.959399000	-0.868757000
1	0.915783000	-2.887684000	-2.189150000
1	1.887970000	-2.956797000	-0.725747000
6	-2.008032000	4.665033000	0.846167000
1	-3.057053000	4.821546000	1.085447000
1	-1.417546000	5.092667000	1.660463000
1	-1.747232000	5.190166000	-0.068940000
6	1.731481000	3.909307000	-5.241441000
1	2.785111000	4.190444000	-5.300942000
1	1.425112000	3.556967000	-6.228758000
1	1.164317000	4.812623000	-5.025172000
6	-3.375386000	-1.860741000	5.014116000
1	-4.413457000	-1.564596000	5.175699000
1	-3.286319000	-2.912191000	5.269200000
1	-2.724221000	-1.298837000	5.685247000
1	-9.080389000	-0.782646000	-2.217816000
